# Exposure to household furry pets influences the gut microbiota of infant at 3–4 months following various birth scenarios

**DOI:** 10.1186/s40168-017-0254-x

**Published:** 2017-04-06

**Authors:** Hein M. Tun, Theodore Konya, Tim K. Takaro, Jeffrey R. Brook, Radha Chari, Catherine J. Field, David S. Guttman, Allan B. Becker, Piush J. Mandhane, Stuart E. Turvey, Padmaja Subbarao, Malcolm R. Sears, James A. Scott, Anita L. Kozyrskyj, the CHILD Study Investigators, M. R. Sears, P. Subbarao, S. S. Anand, M. Azad, A. B. Becker, A. D. Befus, M. Brauer, J. R. Brook, E. Chen, M. Cyr, D. Daley, S. Dell, J. A. Denburg, Q. Duan, T. Eiwegger, H. Grasemann, K. HayGlass, R. Hegele, D. L. Holness, P. Hystad﻿, M. S. Kobor, T. R. Kollmann, A. L. Kozyrskyj, C. Laprise, W. Y. W. Lou, J. Macri, P. M. Mandhane, G. Miller, T. Moraes, P. D. Paré, C. Ramsey, F. Ratjen, A. Sandford, J. A. Scott, J. Scott, F. Silverman, E. Simons, T. Takaro, S. Tebbutt, T. To, S. E. Turvey

**Affiliations:** 1 Department of Pediatrics, University of Alberta, 3-527 Edmonton Clinic Health Academy, 11405–87th Avenue, Edmonton, AB T6G IC9 Canada; 2 Dalla Lana School of Public Health, University of Toronto, Toronto, ON Canada; 3 Faculty of Health Sciences, Simon Fraser University, Burnaby, BC Canada; 4 Department of Obstetrics and Gynecology, University of Alberta, Edmonton, AB Canada; 5 Department of Agricultural, Food and Nutritional Science, University of Alberta, Edmonton, AB Canada; 6 Centre for the Analysis of Genome Evolution and Function, University of Toronto, Toronto, ON Canada; 7 Department of Pediatrics and Child Health, Children’s Hospital Research Institute of Manitoba, University of Manitoba, Winnipeg, MB Canada; 8 Department of Pediatrics, Child & Family Research Institute, BC Children’s Hospital, University of British Columbia, Vancouver, BC Canada; 9 Department of Pediatrics, Hospital for Sick Children, University of Toronto, Toronto, ON Canada; 10 Department of Medicine, McMaster University, Hamilton, ON Canada

**Keywords:** Pets, Infant gut microbiota, Birth, Prenatal, Postnatal

## Abstract

**Background:**

Early-life exposure to household pets has the capacity to reduce risk for overweight and allergic disease, especially following caesarean delivery. Since there is some evidence that pets also alter the gut microbial composition of infants, changes to the gut microbiome are putative pathways by which pet exposure can reduce these risks to health. To investigate the impact of pre- and postnatal pet exposure on infant gut microbiota following various birth scenarios, this study employed a large subsample of 746 infants from the Canadian Healthy Infant Longitudinal Development Study (CHILD) cohort, whose mothers were enrolled during pregnancy between 2009 and 2012. Participating mothers were asked to report on household pet ownership at recruitment during the second or third trimester and 3 months postpartum. Infant gut microbiota were profiled with 16S rRNA sequencing from faecal samples collected at the mean age of 3.3 months. Two categories of pet exposure (i) only during pregnancy and (ii) pre- and postnatally were compared to no pet exposure under different birth scenarios.

**Results:**

Over half of studied infants were exposed to at least one furry pet in the prenatal and/or postnatal periods, of which 8% were exposed in pregnancy alone and 46.8% had exposure during both time periods. As a common effect in all birth scenarios, pre- and postnatal pet exposure enriched the abundance of *Oscillospira* and/or *Ruminococcus* (*P* < 0.05) with more than a twofold greater likelihood of high abundance. Among vaginally born infants with maternal intrapartum antibiotic prophylaxis exposure, *Streptococcaceae* were substantially and significantly reduced by pet exposure (*P* < 0.001, FDRp = 0.03), reflecting an 80% decreased likelihood of high abundance (OR 0.20, 95%CI, 0.06–0.70) for pet exposure during pregnancy alone and a 69% reduced likelihood (OR 0.31, 95%CI, 0.16–0.58) for exposure in the pre- and postnatal time periods. All of these associations were independent of maternal asthma/allergy status, siblingship, breastfeeding exclusivity and other home characteristics.

**Conclusions:**

The impact of pet ownership varies under different birth scenarios; however, in common, exposure to pets increased the abundance of two bacteria, *Ruminococcus* and *Oscillospira*, which have been negatively associated with childhood atopy and obesity.

**Electronic supplementary material:**

The online version of this article (doi:10.1186/s40168-017-0254-x) contains supplementary material, which is available to authorized users.

## Background

Microbial colonization of the infant gastrointestinal tract is an essential process in our life cycle, and microbiota-host interactions during this developmental stage of life have a significant influence on future health. Following birth, the gut microbiota of newborns is characterized by low diversity, dominated by facultative anaerobes such as the Proteobacteria, after which the diversity of strict anaerobes within the Firmicutes and Bacteroidetes phyla increases towards an adult-like profile by 1 year of age [1–3]. Throughout this development, microbial composition is shaped by a number of factors including gestational age, mode of delivery (vaginal vs. caesarean), infant diet (breast milk vs. formula) and antibiotic treatment (direct vs. indirect via mother) [4, 5]. Among several environmental determinants that influence postnatal gut microbial development, rising rates of pet ownership globally have stimulated interest on the impact of household furry pets.

The notion that pets provide an immune benefit to human health stems from the hygiene hypothesis, first proposed by David Strachan in 1989 [6] and subsequently supported by several epidemiological studies [7–12], which attributes risk of allergic disease to overly hygienic environments. With further evidence that gut microbial dysbiosis during infancy is associated with the development of allergic disease, this notion has been revised as the ‘microbiota hypothesis’ [13]. Despite rapid microbial colonization of the gut after birth, environmental microbes in the antenatal and/or early postnatal period represent a critical exposure for early-life immune programming that may have long-term consequences. A pooled analysis of 7000 households documented that dog ownership during the first 2 years of life reduced sensitization to allergens in early childhood, although the evidence for asthma prevention was less clear [14]. In a meta-analysis of six studies that evaluated prenatal exposure to household pets, a lowered risk for allergic disease (atopic dermatitis, asthma) in offspring was found, especially for prenatal dog ownership [10]. Havstad et al. [15] documented lowered IgE levels until 2 years of age following pet exposure during pregnancy, which were not altered by postnatal pet ownership. Further, the prenatal pet association was strongest for children born by caesarean section (CS). Still others have found that postnatal pet ownership modified early-life risk factors for metabolic diseases. In the absence of household pets, Cassidy-Bushrow et al. [16] reported a twofold higher risk of obesity at the age of 2 in CS-delivered infants compared to those born vaginally. However, no association was found between CS delivery and toddler obesity in the presence of pet ownership.

In a pilot study of 24 infants, our group observed higher microbial richness and diversity of the infant gut in the presence of household pets at 3 months of age with under-representation of *Bifidobacteriaceae* and over-representation of *Peptostreptococcaceae* [17]. Nermes et al. [11] found counts of *Bifidobacterium breve* to be lower but *Bifidobacterium longum* to be higher in non-wheezing infants exposed to pets. In their subsequent analysis [18], pet-exposed infants harboured more animal-specific *Bifidobacterium pseudolongum*, indicating the bacterial transfer from pets to infants. Moreover, our previous findings also highlighted that pets can alter house dust microbiota [19]. This study is a follow-up to our previous pilot study, undertaking an evaluation of the influence of household pets on faecal microbial composition at 3–4 months after birth in a large subsample of 746 infants from the Canadian Healthy Infant Longitudinal Development (CHILD) national population-based birth cohort. In this study, we aimed to determine the existence of gut microbial associations with prenatal and/or postnatal pet exposures under different scenarios, independent of siblingship and other covariates.

## Methods

### Study design

**Fig. 1:**
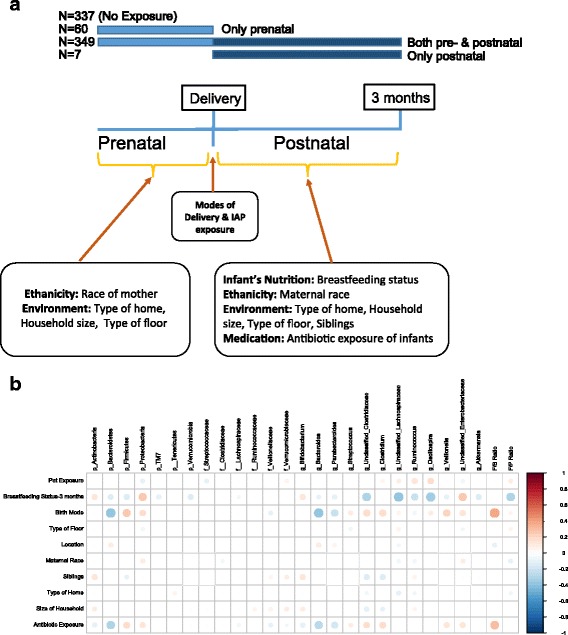
**a** Pet exposure and other covariates (at prenatal and postnatal) that influence the infant gut microbiota. **b** General impact of pet exposure and other covariates on gut microbiota measurements of infants at 3–4 months. *Circle sizes* and *colour intensity* represent the magnitude of correlation. *Red circles* = positive correlations; *blue circles* = negative correlations. Antibiotic exposure of infants was collective consideration of both indirect exposure (maternal IAP) and direct exposure (IV and oral antibiotics)

This study involved a subsample of 804 infants from three study sites (Edmonton, Vancouver and Winnipeg) of the CHILD cohort (www.canadianchildstudy.ca). Mothers of the studied infants were enrolled during pregnancy between 2009 and 2012. The mothers were asked about pet ownership in a standardized questionnaire at recruitment in the second or third trimester of pregnancy and 3 months postpartum. Microbiota analysis was performed on faecal samples collected from infants at 3–4 months, with complete pre- and postnatal pet exposure data (*n* = 753). A pet exposure variable denoting four mutually exclusive categories was created as follows: (1) no pet exposure in the pre- or postnatal periods, (2) only prenatal pet exposure, (3) both pre- and postnatal pet exposure and (4) only postnatal pet exposure (Fig. 1a). Due to the limited number of infants (*n* = 7) in the fourth category, we excluded that category from the analysis, leaving 746 with complete data for subsequent analysis. Table 1 shows demographic characteristics of the studied infants with differential pet exposure status. Data on other covariates were obtained from hospital records (mode of delivery, intrapartum antibiotic prophylaxis (IAP)) or from standardized questionnaires completed by mothers (maternal race, maternal asthma and allergy status during pregnancy, type of home, size of household, type of floor, presence of siblings, breastfeeding status and infant antibiotic exposure before 3 months). Written informed consent was obtained from parents at enrollment. This study was approved by the ethics board at the University of Alberta.

**Table 1:** Population characteristics and associations with exposure to household pets

	Overall	Households with at least 1 furry pet			
		No exposure	Only prenatal	Both pre- and postnatal	*P* value to compare 3 exposure groups
	*N* (%) 746 (100)	*N* (%) 337 (45.2)	*N* (%) 60 (8.0)	*N* (%) 349 (46.8)	
Birth mode					
Vaginal, IAP−	375 (50.3)	171 (45.6)	32 (8.5)	172 (45.9)	0.88
Vaginal, IAP+	176 (23.6)	75 (42.6)	14 (8.0)	87 (49.4)	
Caesarean, scheduled	87 (11.7)	42 (48.3)	4 (4.6)	41 (47.1)	
Caesarean, emergency	108 (14.5)	49 (45.4)	10 (9.3)	49 (45.4)	
Study sites					
Edmonton	216 (29.0)	80 (37.0)	19 (8.8)	117 (54.2)	<0.001
Vancouver	286 (38.3)	158 (55.2)	16 (5.6)	112 (39.2)	
Winnipeg	244 (32.7)	99 (40.6)	25 (10.2)	120 (49.2)	
Maternal race					
Caucasian	561 (75.3)	223 (39.8)	49 (8.7)	289 (51.5)	<0.001
Other	78 (10.5)	38 (48.7)	5 (6.4)	35 (44.9)	
Asian	106 (14.2)	76 (71.7)	6 (5.7)	24 (22.6)	
Maternal asthma during pregnancy					
No	675 (90.6)	307 (45.5)	53 (7.9)	315 (46.7)	0.98
Yes	70 (9.4)	31 (44.3)	6 (8.6)	33 (47.1)	
Maternal allergy during pregnancy					
No	271 (36.6)	133 (49.1)	24 (8.9)	114 (42.1)	0.19
Yes	470 (63.4)	204 (43.4)	36 (7.7)	230 (48.9)	
Type of home					
Single (house)	592 (79.4)	251 (42.4)	53 (9.0)	288 (48.6)	0.007
Multiple (condo/apartment)	154 (20.6)	86 (55.8)	7 (4.5)	61 (39.6)	
Size of household < 3					
No	414 (55.5)	190 (45.9)	34 (8.2)	190 (45.9)	0.86
Yes	332 (44.5)	147 (44.3)	26 (7.8)	159 (47.9)	
Changed house (from 18 weeks of pregnancy to 3 months)					
No	683 (91.6)	309 (45.2)	51 (7.5)	323 (47.3)	0.15
Yes	63 (8.4)	28 (44.4)	9 (14.3)	26 (41.3)	
Type of floor					
Not carpeted	360 (48.3)	156 (43.3)	25 (6.9)	179 (49.7)	0.58
Partially carpeted	235 (31.5)	110 (46.8)	21 (8.9)	104 (44.3)	
Completely carpeted	151 (20.2)	71 (47.0)	14 (9.3)	66 (43.7)	
Siblings					
No	379 (52.6)	162 (42.7)	28 (7.4)	189 (49.9)	0.21
Yes	342 (47.4)	166 (48.5)	28 (8.2)	148 (43.3)	
Antibiotic exposure (0–3 months)^a^					
No	317 (43.8)	142 (44.8)	26 (8.2)	149 (47.0)	0.98
Yes	407 (56.2)	184 (45.2)	32 (7.9)	191 (46.9)	
Breastfeeding status at 3 months					
No	126 (16.9)	46 (36.5)	14 (11.1)	66 (52.4)	0.17
Partial	229 (30.7)	101 (44.1)	18 (7.9)	110 (48.0)	
Exclusive	391 (52.4)	190 (48.6)	28 (7.2)	173 (44.2)	

Comparisons by chi-square test

^a^Antibiotic exposure of infants = collective consideration of both indirect exposure (maternal IAP) and direct exposure (IV and oral antibiotics)

### Faecal microbiota analysis

Faecal samples of infants were collected at the mean age of 3.34 months (range 2.7–4.3) using a standard protocol during a planned home visit. Methods of sample collection, DNA extraction and amplification, 16S rRNA sequencing and taxonomic classification have been previously described [20, 21]. Briefly, samples were refrigerated immediately after collection and during transport and stored at −80 °C until analysis. Genomic DNA was extracted from 80 to 200 mg of stool using the QIAamp DNA Stool Mini kit (Qiagen, Venlo, the Netherlands). The V4 hypervariable region of the bacterial 16S rRNA gene was amplified by PCR using universal bacterial primers: V4-515f: 5′ AAT GAT ACG GCG ACC ACC GAG ATC TAC ACT ATG GTA ATT GTG TGC CAG CMG CCG CGG TAA-3′, V4-806r:5′–CAA GCA GAA GAC GGC ATA CGA GAT XXXXXXXXXXXX AGT CAG TCA GCC GGA CTA CHV GGG TWT CTA AT-3′. For sample multiplexing, reverse primers were barcoded uniquely for each sample (barcoded sequence was denoted in the primer sequence by Xs). Each 25 μl PCR mixture contained 12.5 μl 2x Kapa2G Hotstart mix (Kapa Biosystems, Wilmington, MA), 0.6 μM of both forward and reverse primers and 2 μl genomic DNA (5 ng/μl). PCR amplification consisted of an initial denaturation step for 3 min at 94 °C, followed by 20 cycles of denaturation for 30 s at 94 °C, annealing for 30 s at 50 °C and an extension step for 30 s at 72 °C. PCR reactions for each sample were performed in triplicate with a negative control in each run. One hundred nanograms of pooled PCR product from each sample was concentrated using an Amicon Ultra-4 30K centrifugal filter (Millipore, Billerica, MA, USA), run on a 1.4% agarose gel, extracted and cleaned with the GENE-CLEAN Turbo Kit (MP Biomedicals Inc, Solon, OH, USA).

Pooled PCR amplicons were subjected to paired-end sequencing by Illumina Miseq platform. Using a QIIME pipeline (v1.6.0, qiime.org) [22], forward and reverse reads were assembled using PandaSeq for a final length of 144 bp (unassemblable sequences discarded), demultiplexed and filtered against the GREENGENES reference database (v13.8) [23] to remove all sequences with <60% similarity. Remaining sequences were clustered with Usearch61 at 97% sequence similarity against the GREENGENES database (closed picking algorithm), and taxonomic assignment was achieved using the RDP classifier [24] constrained by GREENGENES. After taxonomic assignment, operational taxonomic units (OTUs) representing bacterial origin were selected, and bacterial OTUs with overall relative abundance below 0.0001 were excluded from subsequence for downstream analyses. To avoid the bias due to variation in sequencing depths among samples, data were rarefied to 13,000 sequences per sample.

### Statistical analysis

With the recommended pipeline in QIIME, relative abundance of bacterial OTUs was summarized at the phylum, family and genus levels. Microbial alpha diversity within samples was calculated with three standard indices (Chao1, Shannon and Simpson). Microbial community differences between samples (beta diversity) were examined by the  permutational multivariate analysis of variance (PERMANOVA) comparison of unweighted UNIFRAC distance matrices, with 1000 permutations. Spearman correlation analyses were performed to address associations between pet exposure, other covariates and microbiota measures, and illustrated using the R package corrplot. Median richness, diversity and relative abundance of dominant taxonomic groups were compared by non-parametric Kruskal-Wallis (KW) test, followed by post hoc comparisons between non-exposed and pet exposure groups using the Mann-Whitney *U* test. As shown in previous reports [20, 25], ratios of specific taxa are commonly evaluated due to the co-existence nature of gut microbiota. We evaluated three ratios: Firmicutes to Bacteroidetes (F/B) ratio, Firmicutes to Proteobacteria (F/P) ratio and *Enterobacteriaceae* to *Bacteroidaceae* (E/B) ratio. Since caesarean birth and maternal IAP are major microbiota disruption exposures [21], we performed our analyses within four different birth scenarios for infants born: (1) vaginally without IAP, (2) vaginally with IAP, (3) by scheduled CS and (4) by emergency CS. To restrict the effects of siblingship and exclusively breastfeeding, comparisons were conducted for specific groups with or without siblings, non-exclusively breastfed infants, as well as non-exclusively breastfed infants without siblings. Independent associations between microbiota abundance and pet exposure were tested by multiple variable logistic regression, with microbiota measures categorised as above and below the median.

To identify discriminative taxonomic biomarkers, the linear discriminant analysis effect size (LEfSE) was determined with a LDA log score cut-off of 2, followed by the Kruskal-Wallis test with the Dunn’s multiple comparison test, using no pet exposure as the reference group. Excluded were infants from non-Caucasian mothers, those with direct antibiotic exposure from birth to 3 months of age and infants exclusively breastfed.

## Results

### Study population and exposures to household furry pets

In this population cohort of 746 infants, less than half of households had no pets (45.2%, *n* = 337), 8% (*n* = 60) of households owned pets only during the index pregnancy (48.3% dog only, 36.1% cat only, 8.3% both dog and cat, and 7.3% other furry pets) and 46.8% (*n* = 349) owned furry pets both in the pregnancy and postnatal time periods (44.1% dog only, 33.8% cat only, 20.1% both dog and cat, 2% other furry pets). Table 1 shows household characteristics for each pet exposure category. Significant differences by pet ownership were found according to study location (*P* < 0.001), maternal race (*P* < 0.001) and type of home (*P* = 0.007).

### Overall community structure of gut microbiota, diversity and richness of gut microbiota

Significant microbial community differences were detected by PERMANOVA by prenatal (pseudo *F* = 2.03, *P* = 0.001), as well as pre- and postnatal exposures (pseudo *F* = 1.51, *P* = 0.005) in all children. Under individual birth scenarios, the impact of any pet exposure was significant only for infants born by emergency CS (pseudo *F* = 2.02, *P* = 0.001) (Additional file 1: Table S1). Overall microbial richness of the infant gut and species richness within the Firmicutes phylum were significantly elevated with pet exposure during pregnancy alone (Additional file 2: Table S2). Upon stratification by birth mode, these trends remained but statistical significance was lost, except for species richness of Firmicutes in vaginally born infants without IAP exposure (Additional file 3: Table S3). Reduced species richness within the Proteobacteria phylum became more statistically significant among infants who were born vaginally without IAP exposure (especially for prenatal exposure alone) and who were born by emergency CS (for both pre- and postnatal exposure) (Additional file 3: Table S3). Although there was no significant impact on overall microbial diversity (Shannon index), pre- and postnatal pet exposure significantly increased the species richness (Chao1) and diversity (Shannon) within the Firmicutes phylum (Additional file 2: Table S2). After stratification by birth scenario, these trends were consistent but were not statistically significant (Additional file 3: Table S3).

### Taxonomic composition of gut microbiota

#### Vaginal with no IAP

Among the dominant phyla, Proteobacteria were under-represented among infants born vaginally with no IAP when pets were present (*P* = 0.005, FDRp = 0.07, Table 2). This reduced abundance was more prominent when pet exposure was solely prenatal, whereas the impact of pre- and postnatal pet ownership became more statistically significant in non-exclusively breastfed infants (Additional file 4: Table S4). In conjunction with depleted Proteobacterial abundance, significant increases to the Firmicutes/Proteobacteria (F/P) ratio were observed by pet exposure (*P* = 0.003, FDRp = 0.05, Table 2). The impact of pre- and postnatal exposure on the F/P ratio also became more significant in non-exclusively breastfed infants (Additional file 4: Table S4).

**Table 2:** Relative abundance of dominant phyla and families in faecal microbiota of infants at 3–4 months, according to birth scenarios and pet exposure

Birth scenarios	Dominant taxa^a^		Pet exposure status (*N* = 746)			
		No exposure 337 (45.2%) Median (IQR)	Only prenatal 60 (8.0%) Median (IQR)	Both pre- and postnatal 349 (46.8%) Median (IQR)	P	FDRp
Vaginal, IAP−		*171 (45.6%)*	*32 (8.5%)*	*172 (45.9%)*		
	Actinobacteria	6.7 (2.4–16.1)	4.3 (1.3–13.2)	7.2 (1.8–18.9)	0.29	0.84
	*Bifidobacteriaceae*	5.4 (1.3–14.6)	5.2 (0.3–14.8)	4.9 (0.99–13.6)	0.96	0.96
	Bacteroidetes	38.6 (2.0–6.9)	37.7 (2.5–73.9)	35.2 (0.87–67.0)	0.71	0.88
	*Bacteroidaceae*	18.1 (0.09–58.4)	1.28 (0.07–35.84)	7.6 (0.07–54.32)	0.41	0.88
	*Porphyromonadaceae*	0.01 (0.00–0.13)	0.01 (0.00–0.60)	0.01 (0.00–0.15)	0.92	0.95
	Firmicutes	17.3 (5.5–32.3)	22.9 (10.1–40.3)	16.2 (7.9–32.3)	0.44	0.88
	*Streptococcaceae*	0.65 (0.21–1.8)	1.1 (0.23–2.8)	0.57 (0.18–1.9)	0.59	0.88
	*Clostridiaceae*	0.33 (0.02–2.1)	0.35 (0.09–1.8)	0.45 (0.06–2.9)	0.52	0.88
	*Lachnospiraceae*	2.6 (0.03–9.4)	4.7 (0.16–12.3)	2.2 (0.04–10.4)	0.59	0.88
	*Rumminococcaceae*	0.09 (0.00–1.10)	0.62 (0.00–2.5)	0.05 (0.00–1.7)	0.55	0.88
	*Vellionellaceae*	4.4 (0.48–17.2)	9.7 (3.4–17.7)	6.2 (1.2–20.3)	0.17	0.72
	Proteobacteria	17.6 (9.7–37.4)	7.2 (2.0–28.6)**	15.5 (7.6–35.4)	0.005	0.07
	*Enterobacteriaceae*	17.0 (6.5–37.8)	12.0 (3.9–47.0)	14.7 (4.8–37.7)	0.61	0.88
	Verrucomicrobia	0.00 (0.00–0.01)	0.00 (0.00–0.01)	0.00 (0.00–0.01)	0.36	0.88
	*Verrucomicrobiaceae*	0.00 (0.00–0.01)	0.00 (0.00–0.01)	0.00 (0.00–0.02)*	0.05	0.45
	F/B ratio	0.47 (0.1–6.2)	0.58 (0.15–12.6)	0.57 (0.15–23.3)	0.67	0.88
	F/P ratio	0.91 (0.29–2.3)	2.5 (0.77–11.6)***	1.0 (0.39–3.4)	0.003	0.05
	E/B ratio	0.84 (0.15–353.7)	21.0 (0.12–333.7)	1.3 (0.12–487.6)	0.92	0.95
Vaginal, IAP+		*75 (42.6%)*	*14 (8.0%)*	*87 (49.4%)*		
	Actinobacteria	2.1 (0.24–10.9)	2.7 (0.52–4.4)	4.0 (0.56–17.8)	0.18	0.72
	*Bifidobacteriaceae*	6.9 (1.3–20.3)	3.7 (1.7–10.9)	3.6 (1.4–14.5)	0.69	0.88
	Bacteroidetes	2.8 (0.07–61.3)	33.7 (0.05–66.9)	21.3 (0.05–69.9)	0.9	0.95
	*Bacteroidaceae*	9.5 (0.08–43.2)	27.6 (0.04–66.5)	35.2 (0.65–65.0)**	0.01	0.12
	*Porphyromonadaceae*	0.01 (0.00–0.28)	0.00 (0.00–0.14)	0.01 (0.00–0.29)	0.52	0.88
	Firmicutes	20.7 (8.2–53.4)	21.1 (5.3–57.9)	17.1 (7.4–46.7)	0.89	0.95
	*Streptococcaceae*	1.0 (0.38–4.1)	0.27 (0.1–0.62)**	0.43 (0.2–0.97)***	<0.001	0.03
	*Clostridiaceae*	0.49 (0.02–2.7)	0.36 (0.1–5.1)	0.29 (0.03–2.0)	0.73	0.88
	*Lachnospiraceae*	1.8 (0.07–10.1)	1.6 (0.03–4.3)	2.4 (0.09–11.4)	0.54	0.88
	*Rumminococcaceae*	0.33 (0.01–1.6)	0.03 (0.00–3.1)	0.12 (0.00–1.2)	0.76	0.88
	*Vellionellaceae*	6.7 (0.7–18.3)	2.6 (0.41–34.4)	3.0 (0.56–11.1)	0.32	0.85
	Proteobacteria	22.9 (11.3-42.5)	26.7 (10.9–52.1)	15.3 (4.5–40.1)	0.06	0.48
	*Enterobacteriaceae*	19.7 (7.2–40.5)	19.2 (6.5–47.2)	13.7 (5.0–33.0)	0.38	0.88
	Verrucomicrobia	0.00 (0.00–0.01)	0.00 (0.00–0.01)	0.00 (0.00–0.01)	0.7	0.88
	*Verrucomicrobiaceae*	0.00 (0.00–0.01)	0.00 (0.00–0.00)	0.00 (0.00–0.01)	0.74	0.88
	F/B ratio	10.3 (0.21–494.0)	0.49 (0.1–1333.3)	1.8 (0.15–819.4)	0.96	0.96
	F/P ratio	0.88 (0.31–2.5)	1.0 (0.14–3.1)	1.4 (0.54–3.6)	0.14	0.63
	E/B ratio	1.7 (0.23–460.4)	2.5 (0.16–1307.1)	0.43 (0.1–64.3)**	0.02	0.21
Caesarean, scheduled		*42 (48.3%)*	*4 (4.6%)*	*41 (47.1%)*		
	Actinobacteria	5.3 (0.89–23.2)	4.7 (0.58–20.9)	9.1 (2.4–17.1)	0.63	0.88
	*Bifidobacteriaceae*	11.3 (3.1–17.0)	0.04 (0.02–17.1)	8.3 (2.4–19.5)	0.26	0.78
	Bacteroidetes	0.13 (0.04–0.62)	30.1 (0.1–61.2)	0.11 (0.05–0.66)	0.58	0.88
	*Bacteroidaceae*	8.0 (0.06–63.1)	3.6 (0.03–28.3)	3.9 (0.11–52.7)	0.56	0.88
	*Porphyromonadaceae*	0.01 (0.00–0.07)	0.00 (0.00–0.00)	0.01 (0.00–0.07)	0.07	0.5
	Firmicutes	31.2 (13.3–55.0)	8.5 (4.5–31.2)	39.2 (16.1–52.8)	0.12	0.63
	*Streptococcaceae*	0.50 (0.3–2.1)	0.15 (0.11–1.2)	0.40 (0.16–1.6)	0.24	0.78
	*Clostridiaceae*	0.77 (0.05–4.4)	3.9 (0.43–16.4)	0.64 (0.09–2.6)	0.74	0.88
	*Lachnospiraceae*	2.1 (0.05–10.2)	5.0 (0.81–28.5)	1.7 (0.05–8.3)	0.71	0.88
	*Rumminococcaceae*	0.08 (0.01–1.8)	3.5 (0.16–9.5)	0.05 (0.00–2.5)	0.41	0.88
	*Vellionellaceae*	3.4 (0.36–10.7)	2.7 (0.93–13.1)	4.9 (0.92–22.7)	0.54	0.88
	Proteobacteria	33.2 (11.4–51.8)	44.4 (13.9–79.9)	34.2 (13.5–50.4)	0.67	0.88
	*Enterobacteriaceae*	16.1 (8.9–35.7)	25.6 (15.9–73.1)	21.8 (8.0–47.1)	0.46	0.88
	Verrucomicrobia	0.00 (0.00–0.01)	0.00 (0.00–0.00)	0.00 (0.00–0.01)	0.33	0.85
	*Verrucomicrobiaceae*	0.00 (0.00–0.00)	0.00 (0.00–0.02)	0.00 (0.00–0.01)	0.23	0.78
	F/B ratio	169.0 (22.8–859.8)	18.2 (0.07–1230.1)	218 (35.0–980.4)	0.33	0.85
	F/P ratio	1.2 (0.35–3.1)	0.41 (0.12–0.75)	1.1 (0.6–3.1)	0.13	0.63
	E/B ratio	1.5 (0.13–353.6)	287 (3.4–907.5)	4.3 (0.19–214.4)	0.57	0.88
Caesarean, emergency		*49 (45.4%)*	*10 (9.3%)*	*49 (45.4%)*		
	Actinobacteria	7.4 (1.4–20.5)	5.6 (3.3–13.5)	6.1 (0.4–16.9)	0.72	0.88
	*Bifidobacteriaceae*	3.5 (0.08–7.0)	28.8 (12.7–71.9)***	3.4 (0.57–18.1)	0.003	0.05
	Bacteroidetes	0.10 (0.03–0.23)	0.15 (0.1–50.6)	0.10 (0.05–1.3)	0.14	0.63
	*Bacteroidaceae*	31.2 (0.07–66.7)	0.19 (0.08–22.1)	5.0 (0.07–67.1)	0.77	0.88
	*Porphyromonadaceae*	0.01 (0.00–0.14)	0.00 (0.00–0.13)	0.01 (0.00–0.04)	0.19	0.72
	Firmicutes	33.3 (19.6–55.1)	48.1 (15.1–67.3)	33.9 (17.5–59.5)	0.74	0.88
	*Streptococcaceae*	0.59 (0.15–1.5)	3.5 (1.7–6.8)**	0.58 (0.21–1.1)	0.003	0.05
	*Clostridiaceae*	0.38 (0.04–7.5)	0.17 (0.01–3.1)	0.70 (0.02–3.7)	0.64	0.88
	*Lachnospiraceae*	2.9 (0.05–7.3)	3.8 (0.05–5.7)	1.6 (0.26–9.1)	0.77	0.88
	*Rumminococcaceae*	0.14 (0.00–2.6)	0.01 (0.01–0.91)	0.08 (0.00–0.58)	0.25	0.78
	*Vellionellaceae*	3.8 (0.56–10.9)	2.3 (0.2–4.8)	2.2 (0.41–13.0)	0.6	0.88
	Proteobacteria	36.7 (19.3–56.4)	27.8 (12.6–38.2)	22.0 (7.7–51.5)*	0.08	0.52
	*Enterobacteriaceae*	20.3 (6.3–40.1)	10.2 (5.7–55.2)	15.0 (8.1–43.1)	0.88	0.95
	Verrucomicrobia	0.00 (0.00–0.01)	0.00 (0.00–0.01)	0.00 (0.00–0.01)	0.81	0.91
	*Verrucomicrobiaceae*	0.00 (0.00–0.01)	0.01 (0.00–0.02)	0.00 (0.00–0.01)	0.14	0.63
	F/B ratio	281 (52.7–1092.0)	362 (0.23–777.5)	252 (10.9–910.5)	0.63	0.88
	F/P ratio	1.2 (0.47–2.8)	1.4 (0.65–4.1)	1.8 (0.65–4.6)	0.22	0.78
	E/B ratio	0.84 (0.09–480.7)	4.3 (0.7–322.8)	10.0 (0.13–336.3)	0.87	0.95

*IQR* interquartile range, *F/B* Firmicutes/Bacteroidetes, *F/P* Firmicutes/Proteobacteria, *E/B Enterobacteriaceae*/*Bacteroidaceae*, *FDR* false discovery rate

Post hoc comparisons between no exposure group and either group of exposure were done by Mann-Whitney *U* test. *P < 0.05; **P < 0.01; ***P < 0.0001

^a^Dominant taxa have overall median relative abundance >1% at −3–4 months; phyla are in the plain text and families are italicized. Comparisons by nonparametric Kruskal-Wallis test with FDR correction for multiple testing

**Fig. 2:**
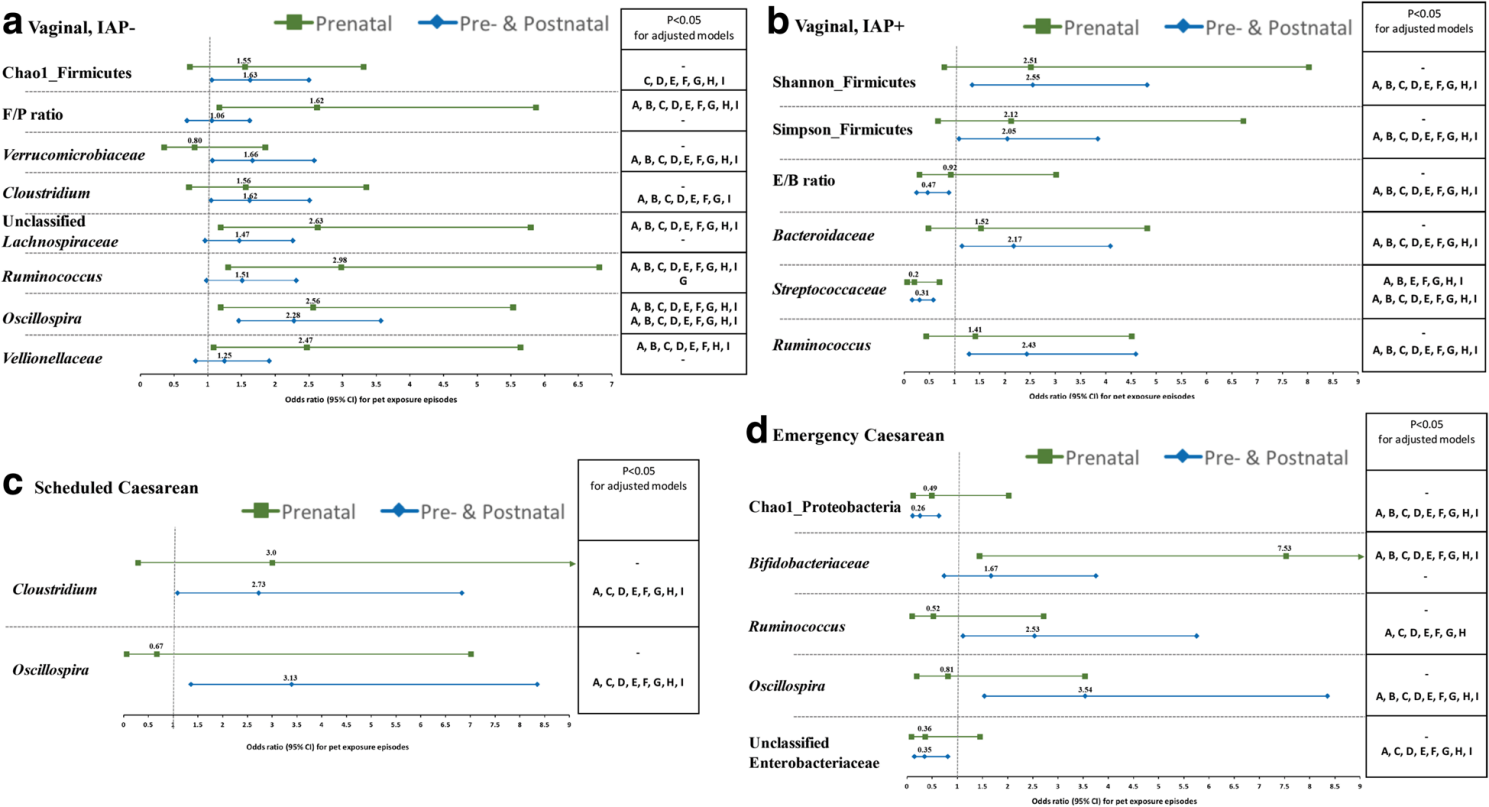
Likelihoods of infant gut microbiota measures at 3–4 months according to pet exposure (parental alone vs. both pre- and postnatal) and various birth scenarios (**a** Vaginal, IAP-, **b** Vaginal, IAP+, **c** Shecduled﻿ Caesarean, and **d** Emerg﻿ency Caesarean), with individual adjustments for potential confounding variables: Model A﻿: location, B: Maternal race, C: Maternal asthma and D: maternal allergy during pregnancy, E: Type of home, F: Moving home, G: Siblingship, H: Antibiotic exposure, and I: Breastfeeding status

With prenatal pet exposure alone, there was a twofold odds for high abundance of *Vellionellaceae* and unclassified *Lachnospiraceae*; high F/P ratios were also more likely (Fig. 2 and Additional file 5: Table S5). These microbiota associations were attenuated when exposure to furry pets continued in the postnatal period. Collectively, pre- and postnatal pet ownership was associated with 1.5-fold increases to high Firmicutes species richness in infants, and high abundance of *Verrucomicrobiaceae* and of genus *Clostridium* (Fig. 2 and Additional file 5: Table S5).

#### Vaginal with IAP

At the family level, median abundance of *Streptococcaceae* were substantially and significantly reduced by prenatal pet exposure (*P* < 0.001, FDRp = 0.03, Table 2). However, the prenatal only association was attenuated in the absence of older siblings at home. Pre- and postnatal pet exposure also enriched the *Bacteroidaceae*, an elevation not seen in non-exclusively breastfed infants (Additional file 4: Table S4).

Twofold increases were found in the odds of high diversity within the Firmicutes and of high abundance with *Bacteroidaceae*. The E/B ratio was reduced by 53% (95%CI 0.25–0.89) and high abundance of *Streptococcaceae* by almost one third (95%CI 0.16–0.58) compared to infants without pet exposure in the pre- and postnatal time periods (Fig. 2 and Additional file 5: Table S5). High *Streptococcaceae* levels were also substantially less likely for infants exposed to pets only during pregnancy (unadjusted OR 0.20, 95%CI 0.06–0.70) (Fig. 2 and Additional file 5: Table S5).

Following exposure to pets during pregnancy and postnatally, a rare member of Proteobacteria, genus *Bilophila*, was depleted after vaginal birth with maternal IAP and no exclusive breastfeeding (*P* = 0.02, log LDA score of 3.5, Additional file 6: Table S6 and Additional file 7: Figure S1).

#### Emergency CS

Enrichment of the median abundance of *Bifidobacteriaceae* was observed in infants born by emergency CS with pet exposure (*P* = 0.003, FDRp = 0.05, Table 2), an association which disappeared in non-exclusively breastfed infants (Additional file 4: Table S4). There was a substantial and significant link between prenatal pet exposure and high abundance of *Bifidobacteriaceae* (unadjusted OR 7.53, 95%CI 1.44–39.50, Additional file 5: Table S5). Concurrently, high species richness within Proteobacteria and high levels of *Enterobacteriaceae* were much less likely to be present (Fig. 2 and Additional file 5: Table S5). When emergency CS was not followed by exclusive breastfeeding, the combined impact of pet exposure during pregnancy and postpartum was reduced abundance of the genus *Citrobacter* (*P* = 0.03, log LDA score of 3.4) and genus *Lactococcus* (*P* = 0.03, log LDA score of 2.5, Additional file 6: Table S6 and Additional file 7: Figure S1).

#### All birth scenarios

At the genus level, *Ruminococcus* and *Oscillospira* were over-represented in infants exposed to pets in all birth scenarios (*P* < 0.05) (Fig. 1b and Table 3). With the exception of vaginal birth with no IAP, these associations were less statistically significant among infants with non-exclusive breastfeeding (Additional file 8: Table S7). Prenatal pet exposure alone was associated with high abundance of *Ruminococcus* (unadjusted OR 2.98, 95%CI 1.30–6.81) and *Oscillospira* (unadjusted OR 2.56, 95%CI 1.19–5.53, Fig. 2 and Additional file 5: Table S5) following vaginal birth with no IAP. Associations with genus *Oscillospira* were unchanged with continuing pet exposure in these infants. More than twofold increases in the odds for high abundance of *Ruminococcus* (unadjusted OR 2.43, 95%CI 1.29–4.59) were also observed following IAP in vaginal birth. In a subgroup of these IAP-exposed infants who were not exclusively breastfed afterwards, the *Ruminococcaceae* population was significantly enriched by prenatal pet exposure alone (*P* = 0.002, and log LDA score of 4.2, Additional file 6: Table S6 and Additional file 7: Figure S1).

**Table 3:** Relative abundance of dominant genera in faecal microbiota of infants at 3–4 months, according to birth scenarios and pet exposure

Birth scenarios	Dominant genera^a^	Pet exposure status (*N* = 746)				
		No exposure 337 (45.17%) Median (IQR)	Only prenatal 60 (8.0%) Median (IQR)	Both pre- and postnatal 349 (46.78%) Median (IQR)	*P*	FDRp
Vaginal, IAP−		*171 (45.6%)*	*32 (8.5%)*	*172 (45.9%)*		
	*Bifidobacterium*	5.3 (1.8–14.9)	2.2 (0.68–13.0)	6.3 (1.6–17.8)	0.21	0.63
	*Bacteroides*	31.2 (0.36–62.4)	29.9 (2.4–62.9)	31.5 (0.61–60.7)	0.66	0.86
	*Parabacteroides*	0.01 (0.00–0.5)	0.16 (0.00–2.9)	0.01 (0.00–0.51)	0.13	0.51
	*Streptococcus*	0.62 (0.13–2.1)	0.46 (0.21–1.4)	0.59 (0.21–1.8)	0.81	0.86
	*Unclassified Clostridiaceae*	0.03 (0.00–0.45)	0.09 (0.00–0.46)	0.07 (0.01–0.49)	0.45	0.81
	*Clostridium*	0.01 (0.00–0.38)	0.01 (0.00–0.29)	0.02 (0.00–0.42)	0.15	0.51
	*Unclassified Lachnospiraceae*	0.02 (0.00–1.6)	0.38 (0.02–4.8)*	0.05 (0.01–1.5)*	0.02	0.16
	*Ruminococcus*	0.02 (0.00–1.2)	0.43 (0.01–1.5)*	0.05 (0.00–2.6)*	0.03	0.21
	*Oscillospira*	0.00 (0.00–0.04)	0.07 (0.00–2.3)*	0.01 (0.00–0.7)***	<0.001	0.04
	*Veillonella*	1.5 (0.16–14.9)	2.9 (0.13–9.1)	1.6 (0.24–7.6)	0.89	0.91
	*Unclassified Enterobacteriaceae*	15.5 (7.0–31.9)	5.4 (1.3–28.4)**	13.3 (4.6–34.8)	0.02	0.16
	*Akkermansia*	0.00 (0.00–0.01)	0.00 (0.00–0.01)	0.00 (0.00–0.01)	0.42	0.81
Vaginal, IAP+		*75 (42.6%)*	*14 (8.0%)*	*87 (49.4%)*		
	*Bifidobacterium*	2.0 (0.06–9.9)	2.2 (0.36–4.1)	3.7 (0.27–16.8)	0.25	0.69
	*Bacteroides*	0.67 (0.05–59.2)	33.7 (0.04–56.3)	12.0 (0.05–69.6)	0.84	0.88
	*Parabacteroides*	0.01 (0.00–0.03)	0.00 (0.00–1.12)	0.00 (0.00–0.02)	0.95	0.95
	*Streptococcus*	0.43 (0.21–1.6)	0.47 (0.12–0.93)	0.59 (0.2–3.6)	0.34	0.81
	*Unclassified Clostridiaceae*	0.08 (0.01–0.88)	0.30 (0.01–3.5)	0.09 (0.01–1.0)	0.49	0.81
	*Clostridium*	0.03 (0.00–0.86)	0.07 (0.02–1.9)	0.04 (0.00–1.0)	0.47	0.81
	*Unclassified Lachnospiraceae*	0.02 (0.00–3.8)	0.66 (0.01–4.1)	0.08 (0.01–3.2)	0.55	0.83
	*Ruminococcus*	0.01 (0.00–0.44)	0.02 (0.00–1.28)	0.03 (0.01–1.88)***	0.004	0.1
	*Oscillospira*	0.00 (0.00–0.56)	0.02 (0.00–2.8)	0.01 (0.00–0.65)	0.66	0.86
	*Veillonella*	3.2 (0.3–13.9)	0.63 (0.19–13.7)	2.7 (0.24–11.8)	0.69	0.86
	*Unclassified Enterobacteriaceae*	20.3 (8.7–37.3)	21.8 (9.8–503)	12.5 (3.6–37.0)*	0.06	0.29
	*Akkermansia*	0.00 (0.00–0.01)	0.00 (0.00–0.01)	0.00 (0.00–0.01)	0.74	0.86
Caesarean, scheduled		*42 (48.3%)*	*4 (4.6%)*	*41 (47.1%)*		
	*Bifidobacterium*	5.0 (0.37–21.6)	3.7 (0.51–19.9)	8.2 (1.3–16.9)	0.76	0.86
	*Bacteroides*	0.08 (0.04–0.44)	30.0 (0.08–61.1)	0.10 (0.05–0.42)	0.49	0.81
	*Parabacteroides*	0.00 (0.00–0.01)	0.00 (0.00–0.04)	0.00 (0.00–0.01)	0.74	0.86
	*Streptococcus*	0.80 (0.29–2.1)	0.46 (0.13–0.75)	1.6 (0.39–3.7)	0.15	0.51
	*Unclassified Clostridiaceae*	0.15 (0.01–1.5)	0.13 (0.02–1.7)	0.40 (0.06–1.6)	0.58	0.84
	*Clostridium*	0.04 (0.01–1.5)	0.16 (0.02–0.59)	0.28 (0.02–1.7)	0.54	0.82
	*Unclassified Lachnospiraceae*	0.04 (0.00–8.7)	2.0 (0.46–22.6)	0.05 (0.00–7.1)	0.42	0.81
	*Ruminococcus*	0.02 (0.00–1.9)	0.00 (0.00–0.01)	0.01 (0.00–1.7)	0.26	0.69
	*Oscillospira*	0.00 (0.00–0.16)	0.00 (0.00–0.01)	0.03 (0.00–0.89)*	0.04	0.24
	*Veillonella*	7.0 (1.04–16.3)	3.6 (1.14–5.41)	6.2 (1.57–29.7)	0.53	0.82
	*Unclassified Enterobacteriaceae*	27.7 (10.1–45.2)	34.9 (13.1–74.7)	30.5 (10.8–47.8)	0.79	0.86
	*Akkermansia*	0.00 (0.00–0.01)	0.00 (0.00–0.00)	0.00 (0.00–0.01)	0.36	0.81
Caesarean, emergency		*49 (45.4%)*	*10 (9.3%)*	*49 (45.4%)*		
	*Bifidobacterium*	6.5 (1.2–18.0)	4.8 (1.9–12.4)	5.3 (0.18–16.8)	0.75	0.86
	*Bacteroides*	0.08 (0.03–0.17)	0.13 (0.05–17.1)	0.09 (0.04–1.3)	0.19	0.61
	*Parabacteroides*	0.00 (0.00–0.01)	0.01 (0.00–0.02)*	0.00 (0.00–0.01)	0.05	0.27
	*Streptococcus*	1.2 (0.5–3.0)	0.64 (0.13–5.1)	0.96 (0.27–2.7)	0.48	0.81
	*Unclassified Clostridiaceae*	0.36 (0.02–3.3)	0.30 (0.01–0.67)	0.31 (0.04–1.6)	0.65	0.86
	*Clostridium*	0.19 (0.02–2.4)	0.04 (0.00–1.1)	0.20 (0.02–1.2)	0.63	0.86
	*Unclassified Lachnospiraceae*	0.03 (0.01–5.2)	0.39 (0.01–1.6)	0.77 (0.01–9.2)	0.41	0.81
	*Ruminococcus*	0.00 (0.00–0.03)	0.00 (0.00–0.33)	0.22 (0.00–4.6)**	0.01	0.16
	*Oscillospira*	0.01 (0.00–0.45)	0.00 (0.00–1.2)	0.19 (0.01–2.8)**	0.02	0.16
	*Veillonella*	9.2 (2.98–26.2)	8.6 (4.3–42.4)	8.1 (0.53–23.8)	0.45	0.81
	*Unclassified Enterobacteriaceae*	34.1 (16.5–54.1)	18.1 (7.5–32.9)	16.4 (6.3–48.4)	0.1	0.44
	*Akkermansia*	0.00 (0.00–0.01)	0.00 (0.00–0.01)	0.00 (0.00–0.01)	0.81	0.86

*IQR* interquartile range, *FDR* false discovery rate

Post hoc comparisons between no exposure group and either group of exposure were done by Mann-Whitney *U* test. *P < 0.05; **P < 0.01; ***P < 0.0001

^a^Dominant genera have overall median relative abundance >1% at 3–4 months; phyla are in plain text and families are in italics. Comparisons by nonparametric Kruskal-Wallis test with FDR correction for multiple testing

Among the few associations found in infants delivered by scheduled CS, a threefold likelihood in high abundance of *Oscillospira* (95%CI 1.27–7.67) was observed with combined prenatal and postnatal pet exposure. High levels of both genus *Oscillospira* (unadjusted OR 3.54, 95%CI 1.54–8.14) and genus *Ruminococcus* (unadjusted OR 2.53, 95%CI 1.11–5.75) were more likely among infants born via emergency CS. In the absence of exclusive breastfeeding after emergency CS, unclassified *Ruminococcaceae* were significantly enriched (*P* = 0.03, log LDA score of 4.0, Additional file 6: Table S6 and Additional file 7: Figure S1).

#### Independence from covariates

To test independence of associations between pet exposure and high microbial diversity, high abundance of microbes and their ratios, we conducted adjusted logistic regression (Fig. 2 and Additional file 5: Table S5). Sequential adjustment for potential confounding variables (Table 1) such as study location, maternal race, maternal history of allergy and asthma during pregnancy, type of home, siblingship, antibiotic exposure and breastfeeding status at 3 months showed that associations between pet ownership and infant gut microbiota were generally robust and independent of these early-life events and environmental exposures. Some associations were moderately attenuated with adjustment, especially for breastfeeding status, maternal race and siblingship (Fig. 2 and Additional file 5: Table S5).

## Discussion

In this general population cohort of 746 Canadian infants, we observed higher overall species richness and changes to taxon abundance in gut microbiota of infants exposed to furry pets during pregnancy or continuing to the postnatal period. These findings are in agreement with our previous report [17] of postnatal pet exposure on 3-month gut microbiota but not with studies in later infancy [26]. Moreover, elevations in microbial species richness in this study were evident with prenatal pet exposure. Since several studies including our own within the same cohort have reported low microbiota richness in early life to be associated with food sensitization and other atopic diseases [20, 27, 28], higher microbial richness with prenatal pet exposure may confer protection against the development of atopy.

Our study revealed that pet exposure significantly increased species richness in the phylum Firmicutes, composed of families like the *Clostridiaceae*, *Lachnospiraceae* and *Ruminococcaceae*. These families of the Firmicutes are obligate anaerobes which reduce the oxidative state of the gut [29]; they are common constituents of the gut microbiota of healthy infants and severely depleted in malnourished infants [30]. In particular, we found *Ruminococcus*, or *Oscillospira*, belonging to the *Ruminococcaceae*, to be more abundant (median levels and levels above the median) among infants exposed to pets pre- and postnatally across all birth scenarios. Associations with ruminococcal abundance above the median were independent of all covariates, but attenuated after adjustment for breastfeeding status and maternal race. Prenatal pet exposure alone was sufficient to produce associations with *Ruminococcus* or *Oscillospira*, even under conditions of undisturbed gut microbiota following vaginal birth and no IAP. Of note, enrichment in faecal *Oscillospira* was among the few changes observed for pet ownership within infants delivered by scheduled CS.

*Oscillospira* is an enigmatic bacterium which has never been isolated in culture, but has been detected by 16S rRNA gene surveys of the human microbiome in association with leanness or lower body mass index in both infants and adults [31–35]. Members of the genus *Oscillospira* are highly heritable, predominate in the lean host and are positively associated with the leanness-promoting bacterium, *Christensenella minuta* [32]. Escober et al. [34] also reported decreasing abundance of *Oscillospira* with obesity in three different geographical regions, despite substantial differences in gut microbial composition. As confirmed by meta-analysis [33], the abundance of *Oscillospira* has also been found to be negatively associated with paediatric inflammatory bowel disease [36]. The health-promoting effects of *Oscillospira* are not fully understood. Unlike *Ruminococcus*, they are not fibre degraders but rather, produce butyrate by relying on fermentation products secreted by other bacterial species or on sugars liberated from host mucins [37]. This is supported by an elegant animal study comparing the microbiota response to fasting across different vertebrates [38]. In this study, *Oscillospira* were observed to be the only genus whose levels increased during fasting, indicating their ability to degrade host glycans such as fucose, sialic acids and glucuronic acid.

Members of *Ruminococcus* have also been detected in the stool of neonates and infants [39] but are reportedly absent in some infants delivered vaginally or by CS [40]. Like the *Oscillospira*, they are also present in dogs and cats [41]. The role of ruminococci in infant health is also poorly understood. Among their noticeable functions, these microbes stimulate the production and degradation of mucin [42], vital to the maintenance of an intact microbiota-mucin barrier. They are also fibre degraders [43] and predominant in formula-fed infants [44, 45]. Yet, ruminococci are still found in breastfed infants and interestingly, their colonization depends on the oligosaccharide content of breast milk [46]. Lastly, they produce ruminococcin A, a bacteriocin which can inhibit various pathogenic species of *Clostridium* [47]. In our previous study within the same cohort, we observed a strong link between low levels of *Ruminococcaceae* and food sensitization at age 1, even after adjustment for major microbiota-disrupting events [20].

Our current study also suggests the potential for pet ownership to assist in reducing the burden of group B *Streptococcus* (GBS) in infants by lowering the abundance of its family, *Streptococcaceae*. According to a recent paper from McCloskey et al. [48], antenatal pet exposure has been linked to reduced cardiovascular risk of infants born to mothers colonized with GBS during pregnancy. In Canada and elsewhere, the major indication for providing IAP is to prevent GBS infection in newborns [49]. Within vaginally born infants with IAP, we found that prenatal pet exposure reduced the abundance of faecal *Streptococcaceae*; this association could not be explained by siblingship, breastfeeding status or other covariates. With mechanisms for microbe interactions to be elucidated, it is conceivable that bacteriocin produced from *Ruminococcus*, a microbe which was more abundant in study infants when *Streptococcaceae* were depleted, inhibits growth of streptococci. However, others have found lowered abundance of *Oscillospira* but elevated levels of *Ruminococcus* to co-occur with a greater abundance of *Streptococcaceae* at 6 months following vaginal GBS colonization in primarily formula-fed infants [50].

Under birth scenarios involving vaginal delivery, Proteobacteria became less abundant in infants with postnatal pet exposure which commenced prenatally. After emergency CS, the following changes with pet exposure were observed for Proteobacteria: reduced species richness, and abundance of *Enterobacteriaceae* and of *Citrobacter*. Pet exposure was also significantly associated with reduced *Enterobacteriaceae* among infants born vaginally without IAP but not exclusively breastfed afterwards. While our findings appear to contradict reports of greater *Escherichia coli* colonization in the vaginal microbiome of pregnant women who own pets [51], the timing of microbial changes in the developmental trajectory of infant microbiota is important to consider. Following vaginal delivery, Proteobacteria (especially *Enterobacteriaceae*) are dominant within 3 months after birth, while Bacteroidetes and Firmicutes become more prevalent as the gut microbiota matures towards an adult-like profile [52]. A bloom of Proteobacteria in the gut can indicate instability in the microbial community [53]; greater abundance (along with a higher abundance of streptococci) in 6-month-old infants has predicted future adiposity [54]. Using the E/B ratio as an indicator for gut microbiota maturity, we previously reported that a higher ratio predicted food sensitization at age 1 [20]; in the current study, pet exposure lowered the E/B ratio in vaginally born infants exposed to IAP. Using another ratio to represent gut microbiota maturity in the current study, pet exposure was linked to a higher F/P ratio following vaginal birth in the absence of maternal IAP. Of note, *Ruminococcus* and *Oscillospira* were also elevated under these circumstances.

Additional discussion of the differential impact of pet exposure on scheduled versus emergency CS is warranted. When compared to scheduled CS, our previous study also reported a distinct microbiota profile in infants born via emergency CS, posited to be a function of the multiplicity of exposures, such as repeated antibiotic treatment and prolonged hospitalization [21]. Here, we also found a greater number of pet-associated microbial changes in infants born by emergency CS. Recurrent antibiotic exposure or hospitalization may render gut microbiota more sensitive to colonization by other microbes [55]. It is also conceivable that pet-induced changes of the maternal microbiome are transmitted to a greater extent during labour prior to an emergency CS than in the absence of labour with scheduled CS.

Our current study has several strengths, including the application of high-throughput deep sequencing to profile gut microbiota in a longitudinal population cohort, with a representative and large sample size. Predominance of Proteobacteria in gut microbiota at 3 months and its higher prevalence in CS-delivered infants were consistent with observations in other birth cohorts. Unlike other studies, our study tested the differential impact of pet exposure according to various birth modes, with the aim of providing more translational information for practitioners. Finally, we implemented statistical modelling and sensitivity analyses to explore whether observed associations were attributable to confounding covariates. On the other hand, the use of 16S rRNA sequencing in our study may have resulted in under-representation of organisms such as bifidobacteria. The sensitivity of this technique also did not allow identification at the species level, which is possible with high-throughput microbial culturomics [56], as well as targeted PCR or phenotypic culturing [57]. Metagenomic sequencing was not conducted, which would enable characterization of the functional properties of microbial changes with pet exposure. Since the majority of households in our study owned at least one dog, a larger sample is required to differentiate the effects of different pet species (e.g. dog and or cat) in future studies.

## Conclusions

With increasing ownership of pets in our modern lifestyle and reports of their beneficial effects, the question of pet ownership is becoming a common one for pregnant women. Our findings highlighted the differential impact of pet exposure on infant gut microbiota following variant birth scenarios; however, in common, the abundance of *Ruminococcus* and *Oscillospira* were found to be increased independent of other factors. In addition, our finding of reduced streptococcal colonization with prenatal pet ownership may lower the risk for childhood metabolic and atopic disease. Further research is needed to link the pet-related microbiota changes with health outcomes of infants in the CHILD cohort, as well as in other populations.

## Additional files

Additional file 1: Table S1. PERMANOVA analysis used to evaluate microbial community differences of infant gut at 3–4 months due to pet exposures following different birth scenarios. (DOCX 35 kb)

Additional file 2: Table S2. Effects of pet exposure on richness and diversity in infant faecal microbiota at 3–4 months. (DOCX 78 kb)

Additional file 3: Table S3. Richness and diversity of infant faecal microbiota at 3–4 months according to birth scenarios and pet exposure. (DOCX 146 kb)

Additional file 4: Table S4. Relative abundance of dominant phyla and families in faecal microbiota of infants belonged to different stratified groups, according to birth scenarios and pet exposure. (DOCX 105 kb)

Additional file 5: Table S5. Crude and adjusted likelihoods of microbiota measurements at 3–4 months according to birth scenarios and pet exposure episodes. (DOCX 126 kb)

Additional file 6: Table S6. Linear discriminant analysis (LDA) scores for differentially abundant bacterial taxa due to pet exposure in formula-fed infants born by Caucasian mothers without prior direct antibiotic exposure until 3 months old following different birth scenarios (*P* < 0.05). (DOCX 54 kb)

Additional file 7: Figure S1. Pet exposure associated changes in gut microbiota of selected infants from Caucasian mothers with no anitobiotic exposure and no exclusi ve breastfeeding at 3 months following different birth scenarios. (PDF 839 kb)

Additional file 8: Table S7. Relative abundance of dominant genera in faecal microbiota of infants belonged to different stratified groups, according to birth scenarios and pet exposure. (DOCX 102 kb)

## Abbreviations

CHILD: Canadian Healthy Infant Longitudinal Development
CS: Caesarean section
IAP: Intrapartum antibiotic prophylaxis
