# Supplementary material for: Exposure to household furry pets influences the gut microbiota of infant at 3–4 months following various birth scenarios
Source: Microbiome. 2017 Apr 6;5:40. doi: 10.1186/s40168-017-0254-x (PMC5382463; doi:10.1186/s40168-017-0254-x)
Supplement: Supplementary file 1 — PERMANOVA analysis used to evaluate microbial community differences of infant gut at 3–4 months due to pet exposures following different birth scenarios. (DOCX 35 kb) [file 40168_2017_254_MOESM1_ESM.docx]

**Table S1. PERMANOVA analysis used to evaluate microbial community differences of infant gut at 3-4 months due to pet exposures following different birth scenarios.**

| **Birth Scenarios** | **Pesudo F** | **P-value** |
| --- | --- | --- |
| Vaginal, IAP- | 1.27 | 0.07 |
| Vaginal, IAP+ | 0.93 | 0.60 |
| Scheduled CS | 0.99 | 0.50 |
| Caesarean-Emergency | 2.02 | 0.001 |
