# Supplementary material for: Exposure to household furry pets influences the gut microbiota of infant at 3–4 months following various birth scenarios
Source: Microbiome. 2017 Apr 6;5:40. doi: 10.1186/s40168-017-0254-x (PMC5382463; doi:10.1186/s40168-017-0254-x)
Supplement: Supplementary file 2 — Effects of pet exposure on richness and diversity in infant faecal microbiota at 3–4 months. (DOCX 78 kb) [file 40168_2017_254_MOESM2_ESM.docx]

**Table S2. Effects of pet exposure on richness and diversity in infant fecal microbiota at 3-4 months.**

|  |  | **Pet exposure episodes (N=746)** | | | |
| --- | --- | --- | --- | --- | --- |
| **Taxa Level** | **Biodiversity Indices** | **No exposure**  337 (45.2%)  Median (IQR) | **Only prenatal**  60 (8%)  Median (IQR) | **Both pre and postnatal**  349 (46.8%)  Median (IQR) | **P** |
| Overall | Chao1 | 189.6 (163.6-222.3) | 204.2 (179.2-241.7)* | 196.4 (170.2-226.8) | 0.04 |
|  | Shannon | 3.1 (2.5-3.5) | 3.0 (2.6-3.5) | 3.0 (2.6-3.5) | 0.98 |
|  | Simpson | 0.77 (0.66-0.83) | 0.77 (0.66-0.83) | 0.76 (0.65-0.84) | 0.89 |
| Actinobacteria | Chao1 | 17.5 (11.0-22.7) | 17.2 (12.1-22.8) | 18.5 (12.0-23.0) | 0.61 |
|  | Shannon | 1.4 (1.1-1.8) | 1.6 (1.1-1.9) | 1.4 (1.1-1.9) | 0.50 |
|  | Simpson | 0.41 (0.31-0.59) | 0.48 (0.33-0.58) | 0.43 (0.32-0.59) | 0.41 |
| Bacteroidetes | Chao1 | 34.0 (8.0-68.5) | 51.6 (11.3-89.8) | 35.3 (9.0-69.3) | 0.15 |
|  | Shannon | 1.7 (1.0-2.4) | 1.9 (1.3-2.3) | 1.8 (1.0-2.3) | 0.45 |
|  | Simpson | 0.56 (0.31-0.73) | 0.57 (0.39-0.72) | 0.56 (0.27-0.72) | 0.84 |
| Firmicutes | Chao1 | 68.0 (48.5-91.9) | 79.7 (59.0-98.56)* | 75.0 (55.9-93.4)* | 0.02 |
|  | Shannon | 2.5 (1.9-3.0) | 2.7 (1.9-3.2) | 2.6 (2.07-3.1)* | 0.05 |
|  | Simpson | 0.71 (0.56-0.8) | 0.71 (0.55-0.83) | 0.74 (0.58-0.82) | 0.17 |
| Proteobacteria | Chao1 | 62.1 (45.5-75.3) | 58.9 (35.3-71.6) | 55.5 (39.5-74.6) | 0.10 |
|  | Shannon | 1.5 (1.2-1.9) | 1.5 (1.2-1.9) | 1.4 (1.1-1.9) | 0.64 |
|  | Simpson | 0.37 (0.29-0.55) | 0.41 (0.31-0.56) | 0.38 (0.29-0.55) | 0.49 |

Richness and diversity indices calculated at OTU level and comparisons by nonparametric Kruskal-Wallis test.

IQR, interquartile range.

Post-hoc comparisons between no exposure group and either group of exposure were done by Mann-Whitney U test. * *P<0.05*, ***P<0.01*, ****P<0.0001*.
