# Supplementary material for: Exposure to household furry pets influences the gut microbiota of infant at 3–4 months following various birth scenarios
Source: Microbiome. 2017 Apr 6;5:40. doi: 10.1186/s40168-017-0254-x (PMC5382463; doi:10.1186/s40168-017-0254-x)
Supplement: Supplementary file 3 — Richness and diversity of infant faecal microbiota at 3–4 months according to birth scenarios and pet exposure. (DOCX 146 kb) [file 40168_2017_254_MOESM3_ESM.docx]

**Table S3. Richness and diversity of infant fecal microbiota at 3-4 months according to birth scenarios and pet exposure.**

| **Birth Scenarios** | **Biodiversity Indices** | **Pet exposure episodes (N=746)** | | | | |
| --- | --- | --- | --- | --- | --- | --- |
|  |  | **No exposure**  337 (45.2%)  Median (IQR) | **Only prenatal**  60 (8%)  Median (IQR) | **Both pre and postnatal**  349 (46.8%)  Median (IQR) | **P** |  |
| Vaginal  IAP- | Chao1 | 194.1 (169.7-227.6) | 212.0 (174.0-245.3) | 202.1 (177.0-231.4) | 0.17 |  |
|  | Shannon | 3.2 (2.7-3.6) | 3.1 (2.4-3.7) | 3.1 (2.6-3.6) | 1.00 |  |
|  | Simpson | 0.78 (0.68-0.83) | 0.76 (0.65-0.83) | 0.77 (0.67-0.84) | 0.78 |  |
|  | Chao1_Bacteroidetes | 53.8 (21.0-86.0) | 79.0 (45.4-93.7) | 50.9 (17.1-90.2) | 0.27 |  |
|  | Shannon_Bacteroidetes | 1.7 (1.0-2.5) | 2.0 (1.3-2.5) | 1.7 (1.0-2.3) | 0.29 |  |
|  | Simpson_Bacteroidtes | 0.5 (0.24-0.71) | 0.56 (0.34-0.73) | 0.51 (0.27-0.7) | 0.45 |  |
|  | Chao1_Firmicutes | 60.3 (44.8-86.1) | 72.1 (56.8-98.4) | 72.9 (51.5-86.5)* | 0.04 |  |
|  | Shannon_Firmicutes | 2.4 (1.9-3.0) | 2.6 (1.9-3.2) | 2.6 (2.0-3.2) | 0.22 |  |
|  | Simpson_Firmictues | 0.7 (0.54-0.8) | 0.7 (0.57-0.81) | 0.74 (0.57-0.82) | 0.39 |  |
|  | Chao1_Proteobacteria | 57.3 (43.3-73.4) | 43.4 (26.7-65.0)* | 55.3 (38.4-72.9) | 0.06 |  |
|  | Shannon_Proteobacteria | 1.5 (1.1-1.9) | 1.6 (1.2-1.9) | 1.5 (1.1-1.9) | 0.53 |  |
|  | Simpson_Proteobacteria | 0.36 (0.28-0.56) | 0.45 (0.31-0.64) | 0.39 (0.29-0.57) | 0.23 |  |
| Vaginal  IAP+ | Chao1 | 185.7 (155.1-220.1) | 196.2 (181.9-237.5) | 189.1 (157.7-224.0) | 0.38 |  |
|  | Shannon | 3.0 (2.5-3.3) | 2.8 (2.5-3.5) | 2.9 (2.4-3.4) | 0.68 |  |
|  | Simpson | 0.77 (0.63-0.82) | 0.74 (0.64-0.83) | 0.72 (0.62-0.83) | 0.33 |  |
|  | Chao1_Bacteroidetes | 37.0 (8.0-71.0) | 25.5 (5.3-89.8) | 33.2 (8.0-62.0) | 0.96 |  |
|  | Shannon_Bacteroidetes | 1.6 (1.2-2.5) | 1.7 (1.2-2.3) | 1.6 (0.93-2.3) | 0.30 |  |
|  | Simpson_Bacteroidtes | 0.63 (0.31-0.75) | 0.56 (0.37-0.68) | 0.54 (0.22-0.74) | 0.25 |  |
|  | Chao1_Firmicutes | 65.5 (46.0-87.5) | 89.5 (57.3-103.1) | 71.3 (50.5-89.4) | 0.23 |  |
|  | Shannon_Firmicutes | 2.4 (1.8-2.7) | 2.8 (1.8-3.6) | 2.7 (2.0-3.2) | 0.10 |  |
|  | Simpson_Firmictues | 0.69 (0.55-0.77) | 0.76 (0.5-0.85) | 0.76 (0.55-0.83) | 0.15 |  |
|  | Chao1_Proteobacteria | 61.1 (45.5-73.3) | 63.0 (52.6-82.8) | 51.8 (39.0-74.1) | 0.15 |  |
|  | Shannon_Proteobacteria | 1.4 (1.1-2.0) | 1.5 (1.1-1.9) | 1.5 (1.2-1.9) | 0.99 |  |
|  | Simpson_Proteobacteria | 0.36 (0.29-0.56) | 0.41 (0.27-0.51) | 0.41 (0.29-0.55) | 0.83 |  |
| Caesarean-scheduled | Chao1 | 183.4 (158.1-201.5) | 188.2 (155.5-239.0) | 190.1 (171.3-210.0) | 0.41 |  |
|  | Shannon | 3.0 (2.4-3.4) | 3.2 (2.5-3.4) | 3.2 (2.8-3.4) | 0.72 |  |
|  | Simpson | 0.75 (0.59-0.83) | 0.77 (0.58-0.83) | 0.78 (0.68-0.83) | 0.80 |  |
|  | Chao1_Bacteroidetes | 11.0 (4.0-22.0) | 35.2 (13.6-82.4) | 12.7 (4.0-30.5) | 0.16 |  |
|  | Shannon_Bacteroidetes | 1.6 (0.89-2.2) | 1.9 (1.6-2.3) | 1.8 (1.3-2.6) | 0.29 |  |
|  | Simpson_Bacteroidtes | 0.56 (0.31-0.72) | 0.61 (0.42-0.72) | 0.64 (0.38-0.78) | 0.43 |  |
|  | Chao1_Firmicutes | 80.7 (56.1-99.8) | 64.2 (59.7-77.9) | 83.6 (68.0-102.5) | 0.25 |  |
|  | Shannon_Firmicutes | 2.6 (2.1-3.1) | 3.2 (2.3-3.2) | 2.6 (2.2-3.0) | 0.50 |  |
|  | Simpson_Firmictues | 0.74 (0.61-0.82) | 0.83 (0.6-0.84) | 0.76 (0.63-0.81) | 0.62 |  |
|  | Chao1_Proteobacteria | 65.3 (48.8-78.5) | 77.6 (31.8-93.3) | 64.1 (49.1-80.5) | 0.81 |  |
|  | Shannon_Proteobacteria | 1.5 (1.2-1.8) | 1.5 (0.82-2.1) | 1.4 (1.1-1.8) | 0.27 |  |
|  | Simpson_Proteobacteria | 0.4 (0.3-0.5) | 0.4 (0.2-0.58) | 0.33 (0.27-0.46) | 0.54 |  |
| Caesarean-emergency | Chao1 | 190.4 (154.4-217.1) | 205.6 (164.7-217.4) | 194 (164.0-225.3) | 0.71 |  |
|  | Shannon | 3.2 (2.5-3.6) | 3.0 (2.8-3.5) | 3.1 (2.6-3.7) | 0.95 |  |
|  | Simpson | 0.77 (0.64-0.84) | 0.77 (0.67-0.83) | 0.78 (0.68-0.86) | 0.85 |  |
|  | Chao1_Bacteroidetes | 10.0 (4.7-24.0) | 16.3 (6.0-55.6) | 10.0 (6.0-32.6) | 0.29 |  |
|  | Shannon_Bacteroidetes | 2.0 (1.5-2.5) | 2.1 (1.3-2.4) | 2.0 (1.2-2.4) | 0.88 |  |
|  | Simpson_Bacteroidtes | 0.67 (0.52-0.78) | 0.63 (0.43-0.76) | 0.67 (0.41-0.76) | 0.75 |  |
|  | Chao1_Firmicutes | 82.3 (57.1-113.1) | 88.88 (64.2-109.9) | 87.09 (66.2-115.4) | 0.76 |  |
|  | Shannon_Firmicutes | 2.7 (2.1-3.1) | 2.4 (1.9-3.0) | 2.8 (2.1-3.2) | 0.70 |  |
|  | Simpson_Firmictues | 0.76 (0.6-0.82) | 0.68 (0.51-0.77) | 0.73 (0.63-0.81) | 0.45 |  |
|  | Chao1_Proteobacteria | 69.2 (59.4-83.6) | 68.6 (50.3-76.9) | 57.1 (40.1-75.5)* | 0.06 |  |
|  | Shannon_Proteobacteria | 1.6 (1.4-2.0) | 1.4 (1.2-1.9) | 1.4 (1.2-2.0) | 0.31 |  |
|  | Simpson_Proteobacteria | 0.41 (0.32-0.58) | 0.36 (0.31-0.52) | 0.36 (0.29-0.57) | 0.78 |  |

Richness and diversity indices calculated at OTU level for overall as well as under each phylum. Comparisons by nonparametric Kruskal-Wallis test.

IQR, interquartile range.

Post-hoc comparisons between no exposure group and either group of exposure were done by Mann-Whitney U test. * *P<0.05*, ***P<0.01*, ****P<0.0001*.
