# Supplementary material for: Exposure to household furry pets influences the gut microbiota of infant at 3–4 months following various birth scenarios
Source: Microbiome. 2017 Apr 6;5:40. doi: 10.1186/s40168-017-0254-x (PMC5382463; doi:10.1186/s40168-017-0254-x)
Supplement: Supplementary file 4 — Relative abundance of dominant phyla and families in faecal microbiota of infants belonged to different stratified groups, according to birth scenarios and pet exposure. (DOCX 105 kb) [file 40168_2017_254_MOESM4_ESM.docx]

**Table S4. Relative abundance of dominant phyla and families in fecal microbiota of infants belonged to different stratified groups, according to birth scenarios and pet exposure.**

| **Birth Scenarios** | **Dominant Taxa^#^** | **No Siblings** | | | | **No Exclusive Breastfeeding** | | | | **No Siblings and No Exclusive Breastfeeding** | | | |
| --- | --- | --- | --- | --- | --- | --- | --- | --- | --- | --- | --- | --- | --- |
|  |  | **No exposure** | **Only prenatal** | **Both pre and postnatal** | **P** | **No exposure** | **Only prenatal** | **Both pre and postnatal** | **P** | **No exposure** | **Only prenatal** | **Both pre and postnatal** | **P** |
|  |  | 162 (42.6%)  Median  (IQR) | 29 (7.6%)  Median  (IQR) | 189 (49.8%)  Median  (IQR) |  | 147 (41.4%)  Median  (IQR) | 32 (9.0%)  Median  (IQR) | 176 (49.6%)  Median  (IQR) |  | 70 (38.7%)  Median  (IQR) | 12 (6.6%)  Median  (IQR) | 99 (54.7%)  Median  (IQR) |  |
| Vaginal IAP- | Proteobacteria | 20.7  (11.3-40.0) | 7.3  (3.6-28.1)* | 15.7  (7.7-35.1) | 0.04 | 16.1  (7.0-33.4) | 2.4  (1.2-10.1)** | 11.3  (5.2-25.7)** | <0.001 | 15.9  (9.2-33.1) | 2.3  (0.92-10.6)** | 10.4  (4.7-22.5)** | 0.002 |
|  | *Verrucomicrobiaceae* | 0  (0-0.01) | 0  (0-0) | 0  (0-0.01) | 0.07 | 0  (0-0.01) | 0  (0-0.02) | 0  (0-0.02) | 0.14 | 0  (0-0) | 0  (0-0.12) | 0  (0-0.02) | 0.01 |
|  | F/P ratio | 1.1  (0.33-2.2) | 2.4  (0.58-10.8)* | 0.86 (0.33-3.4) | 0.18 | 1.2  (0.33-3.8) | 8.5  (1.7-17.1)** | 1.5  (0.64-4.4)** | 0.001 | 1.6  (0.35-3.8) | 9.7  (3.1-17.7)*** | 1.8  (0.76-4.7)** | 0.003 |
| Vaginal IAP+ | *Bacteroidaceae* | 2.2  (0.08-29.1) | 41.2  (0.03-72.3) | 46.0  (0.36-65.0)* | 0.01 | 20.6  (0.04-49.3) | 44.0  (0.03-72.3) | 28.0  (0.19-61.6) | 0.37 | 2.2  (0.02-30.2) | 44.0  (0.02-80.6) | 32.7  (0.2-66.5) | 0.2 |
|  | *Streptococcaceae* | 0.99  (0.41-4.1) | 0.43  (0.02-2.9) | 0.43  (0.21-0.97)* | 0.03 | 1.7  (0.54-4.7) | 0.28  (0.16-0.62)* | 0.5  (0.21-1.7)* | 0.01 | 2.0  (0.5-5.0) | 0.43  (0.12-1.7) | 0.51  (0.23-1.7)* | 0.03 |
|  | E/B ratio | 8.2  (0.39-428.2) | 0.46  (0.16-3179.1) | 0.36  (0.13-89.7)* | 0.03 | 1.3  (0.19-680.4) | 0.46  (0.17-3179.1) | 0.49  (0.14-90.8) | 0.53 | 2.6  (0.43-711.3) | 0.46  (0.16-3924.5) | 0.4  (0.14-144.1) | 0.28 |
| Caesarean-emergency | *Bifidobacteriaceae* | 3.3  (0.05-7.6) | 38.4  (16.8-73.6)** | 5.3  (1.2-18.7) | 0.001 | 3.8  (0.32-7.9) | 16.4  (0.85-51.0) | 5.8  (0.39-20.3) | 0.44 | 3.7 (0.04-7.7) | 10.4  (0.45-66.9) | 5.8  (0.39-20.3)* | 0.55 |
|  | *Streptococcaceae* | 0.73  (0.19-1.5) | 4.5 (1.01-6.5)* | 0.54  (0.21-0.95) | 0.01 | 1.0  (0.13-1.7) | 2.0  (0.89-2.4) | 0.69  (0.35-2.3) | 0.35 | 1.0  (0.22-1.5) | 1.5  (0.83-2.4) | 0.69  (0.35-2.3) | 0.45 |
|  | Proteobacteria | 35.8  (20.7-57.1) | 34.7  (17.0-37.8) | 16.0  (6.3-46.7)* | 0.1 | 29.0  (19.0-50.5) | 34.7  (8.1-37.5) | 14.0  (5.4-27.4)** | 0.05 | 22.9  (16.7-48.6) | 36.0  (18.7-37.7) | 14.0  (5.4-27.4)** | 0.07 |

IQR, interquartile rage; F/P, Firmicutes/Proteobacteria; E/B, Enterobacteriaceae/Bacteroidaceae.

^#^Dominant taxa have overall median relative abundance >1% at -3-4 months; phyla are in the plain text and families are italicized. Comparisons by nonparametric Kruskal-Wallis test.

Post-hoc comparisons between no exposure group and either group of exposure were done by Mann-Whitney U test. * *P<0.05*, ***P<0.01*, ****P<0.0001*.
