# Supplementary material for: Exposure to household furry pets influences the gut microbiota of infant at 3–4 months following various birth scenarios
Source: Microbiome. 2017 Apr 6;5:40. doi: 10.1186/s40168-017-0254-x (PMC5382463; doi:10.1186/s40168-017-0254-x)
Supplement: Supplementary file 5 — Crude and adjusted likelihoods of microbiota measurements at 3–4 months according to birth scenarios and pet exposure episodes. (DOCX 126 kb) [file 40168_2017_254_MOESM5_ESM.docx]

**Table S5. Crude and adjusted likelihoods of microbiota measurements at 3-4 months according to birth scenarios and pet exposure episodes.**

| **Delivery Mode** | **Microbiota Measurement**  **Model Adjustment** | **Prenatal Pet Exposure Alone**  **OR (95% CI)** | **Pet exposure during both pre and postnatal**  **OR (95% CI)** |
| --- | --- | --- | --- |
| Vaginal  IAP- | *Chao1_Firmicutes* (Crude OR)  Adjusted for location  Adjusted for maternal race  Adjusted for maternal asthma during pregnancy  Adjusted for maternal allergy during pregnancy  Adjusted for type of home  Adjusted for moving home  Adjusted for present of siblings  Adjusted for antibiotic exposure^#^  Adjusted for breastfeeding status-3 months | 1.55 (0.73-3.31)  1.31 (0.60-2.83)  1.45 (0.68-3.12)  1.58 (0.74-3.37)  1.55 (0.73-3.31)  1.53 (0.72-3.27)  1.44 (0.67-3.10)  1.48 (0.67-3.27)  1.42 (0.65-3.11)  1.33 (0.60-2.95) | 1.63 (1.06-2.50)*  1.53 (0.99-2.36)  1.49 (0.96-2.32)  1.63 (1.06-2.51)*  1.67 (1.08-2.56)*  1.62 (1.05-2.48)*  1.63 (1.06-2.50)*  1.68 (1.09-2.59)*  1.68 (1.08-2.61)*  1.54 (0.99-2.41) |
|  | *F/P ratio* (Crude OR)  Adjusted for location  Adjusted for maternal race  Adjusted for maternal asthma during pregnancy  Adjusted for maternal allergy during pregnancy  Adjusted for type of home  Adjusted for moving home  Adjusted for present of siblings  Adjusted for antibiotic exposure^#^  Adjusted for breastfeeding status-3 months | 2.62 (1.17-5.87)*  2.38 (1.05-5.39)*  2.56 (1.14-5.76)*  2.60 (1.16-5.83)*  2.62 (1.17-5.87)*  2.58 (1.15-5.80)*  2.57 (1.14-5.78)*  3.21 (1.34-7.66)*  2.84 (1.23-6.60)*  2.31 (1.00-5.35) | 1.06 (0.69-1.62)  1.01 (0.66-1.55)  1.03 (0.67-1.60)  1.06 (0.69-1.62)  1.05 (0.69-1.61)  1.05 (0.69-1.61)  1.06 (0.69-1.62)  1.10 (0.72-1.70)  1.16 (0.75-1.79)  0.94 (0.60-1.47) |
|  | *Verrucomicrobiaceae* (Crude OR)  Adjusted for location  Adjusted for maternal race  Adjusted for maternal asthma during pregnancy  Adjusted for maternal allergy during pregnancy  Adjusted for type of home  Adjusted for moving home  Adjusted for present of siblings  Adjusted for antibiotic exposure^#^  Adjusted for breastfeeding status-3 months | 0.80 (0.35-1.85)  0.82 (0.35-1.91)  0.86 (0.37-1.98)  0.79 (0.34-1.83)  0.80 (0.35-1.85)  0.80 (0.35-1.86)  0.75 (0.32-1.75)  0.95 (0.40-2.23)  0.88 (0.38-2.06)  0.83 (0.36-1.91) | 1.66 (1.07-2.58)*  1.69 (1.09-2.64)*  1.81 (1.15-2.86)*  1.66 (1.07-2.58)*  1.65 (1.06-2.56)*  1.67 (1.07-2.58)*  1.66 (1.07-2.58)*  1.67 (1.06-2.61)*  1.62 (1.03-2.55)*  1.69 (1.09-2.63)* |
|  | *Clostridium* (Crude OR)  Adjusted for location  Adjusted for maternal race  Adjusted for maternal asthma during pregnancy  Adjusted for maternal allergy during pregnancy  Adjusted for type of home  Adjusted for moving home  Adjusted for present of siblings  Adjusted for antibiotic exposure^#^  Adjusted for breastfeeding status-3 months | 1.56 (0.72-3.35)  1.71 (0.79-3.73)  1.60 (0.74-3.48)  1.57 (0.73-3.39)  1.56 (0.72-3.35)  1.60 (0.74-3.46)  1.51 (0.70-3.28)  1.38 (0.61-3.14)  1.58 (0.72-3.50)  1.47 (0.68-3.19) | 1.62 (1.05-2.51)*  1.70 (1.09-2.64)*  1.71 (1.09-2.69)*  1.62 (1.05-2.52)*  1.64 (1.06-2.54)*  1.64 (1.06-2.55)*  1.62 (1.05-2.51)*  1.57 (1.00-2.47)*  1.53 (0.97-2.42)  1.56 (1.01-2.43)* |
|  | *Unclassified Lachnospiraceae* (Crude OR)  Adjusted for location  Adjusted for maternal race  Adjusted for maternal asthma during pregnancy  Adjusted for maternal allergy during pregnancy  Adjusted for type of home  Adjusted for moving home  Adjusted for present of siblings  Adjusted for antibiotic exposure^#^  Adjusted for breastfeeding status-3 months | 2.63 (1.19-5.79)*  2.30 (1.03-5.12)*  2.54 (1.15-5.81)*  2.56 (1.16-5.66)*  2.63 (1.19-5.79)*  2.59 (1.17-5.72)*  2.66 (1.20-5.89)*  2.62 (1.15-5.99)*  2.28 (1.02-5.11)*  2.43 (1.03-5.70)* | 1.47 (0.96-2.26)  1.40 (0.91-2.15)  1.42 (0.92-2.20)  1.47 (0.96-2.25)  1.47 (0.96-2.26)  1.46 (0.96-2.25)  1.48 (0.96-2.26)  1.51 (0.98-2.32)  1.51 (0.97-2.34)  1.40 (0.88-2.23) |
|  | *Ruminococcus* (Crude OR)  Adjusted for location  Adjusted for maternal race  Adjusted for maternal asthma during pregnancy  Adjusted for maternal allergy during pregnancy  Adjusted for type of home  Adjusted for moving home  Adjusted for present of siblings  Adjusted for antibiotic exposure^#^  Adjusted for breastfeeding status-3 months | 2.98 (1.30-6.81)*  2.85 (1.22-6.62)*  2.84 (1.23-6.52)*  2.92 (1.28-6.69)*  3.03 (1.32-6.97)*  2.86 (1.25-6.56)*  3.04 (1.32-7.00)*  3.23 (1.34-7.74)*  2.64 (1.13-6.13)*  2.79 (1.20-6.48)* | 1.51 (0.98-2.31)  1.46 (0.95-2.25)  1.42 (0.92-2.21)  1.50 (0.98-2.30)  1.45 (0.95-2.23)  1.48 (0.96-2.27)  1.51 (0.99-2.31)  1.59 (1.03-2.45)*  1.46 (0.94-2.27)  1.46 (0.94-2.25) |
|  | *Oscillospira* (Crude OR)  Adjusted for location  Adjusted for maternal race  Adjusted for maternal asthma during pregnancy  Adjusted for maternal allergy during pregnancy  Adjusted for type of home  Adjusted for moving home  Adjusted for present of siblings  Adjusted for antibiotic exposure^#^  Adjusted for breastfeeding status-3 months | 2.56 (1.19-5.53)*  2.34 (1.08-5.10)*  2.63 (1.21-5.71)*  2.52 (1.17-5.45)*  2.61 (1.20-5.67)*  2.51 (1.16-5.42)*  2.64 (1.21-5.73)*  2.76 (1.23-6.20)*  2.26 (1.02-5.02)*  2.34 (1.03-5.32)* | 2.28 (1.46-3.57)*  2.20 (1.40-3.46)*  2.37 (1.49-3.77)*  2.28 (1.45-3.56)*  2.20 (1.40-3.46)*  2.26 (1.44-3.54)*  2.29 (1.46-3.58)*  2.35 (1.48-3.71)*  2.33 (1.47-3.70)*  2.29 (1.42-3.68)* |
|  | *Vellionellaceae* (Crude OR)  Adjusted for location  Adjusted for maternal race  Adjusted for maternal asthma during pregnancy  Adjusted for maternal allergy during pregnancy  Adjusted for type of home  Adjusted for moving home  Adjusted for present of siblings  Adjusted for antibiotic exposure^#^  Adjusted for breastfeeding status-3 months | 2.47 (1.08-5.64)*  2.47 (1.08-5.69)*  2.49 (1.09-5.73)*  2.42 (1.05-5.53)*  2.47 (1.08-5.64)*  2.35 (1.03-5.40)*  2.42 (1.05-5.55)*  2.24 (0.96-5.24)  2.64 (1.11-6.38)*  2.36 (1.03-5.42)* | 1.25 (0.82-1.91)  1.25 (0.82-1.92)  1.25 (0.81-1.94)  1.24 (0.81-1.90)  1.23 (0.81-1.89)  1.22 (0.80-1.87)  1.25 (0.82-1.91)  1.31 (0.85-2.02)  1.29 (0.83-2.00)  1.21 (0.79-1.86) |
| Vaginal  IAP+ | *Shannon_Firmicutes* (Crude OR)  Adjusted for location  Adjusted for maternal race  Adjusted for maternal asthma during pregnancy  Adjusted for maternal allergy during pregnancy  Adjusted for type of home  Adjusted for moving home  Adjusted for present of siblings  Adjusted for breastfeeding status-3 months | 2.51 (0.79-8.02)  2.32 (0.72-7.52)  2.44 (0.75-7.91)  2.51 (0.78-8.06)  2.57 (0.83-8.24)  2.44 (0.76-7.83)  2.51 (0.79-8.03)  2.47 (0.77-7.93)  2.68 (0.77-9.29) | 2.55 (1.35-4.82)*  2.44 (1.28-4.63)*  2.51 (1.32-4.77)*  2.51 (1.32-4.77)*  2.57 (1.35-4.88)*  2.54 (1.34-4.80)*  2.55 (1.34-4.85)*  2.32 (1.22-4.43)*  2.95 (1.46-5.96)* |
|  | *Simpson_Firmicutes* (Crude OR)  Adjusted for location  Adjusted for maternal race  Adjusted for maternal asthma during pregnancy  Adjusted for maternal allergy during pregnancy  Adjusted for type of home  Adjusted for moving home  Adjusted for present of siblings  Adjusted for breastfeeding status-3 months | 2.12 (0.67-6.72)  1.96 (0.61-6.36)  2.26 (0.70-7.35)  2.10 (0.66-6.70)  2.12 (0.67-6.74)  2.04 (0.64-6.51)  2.08 (0.65-6.62)  2.10 (0.66-6.70)  2.12 (0.61-7.20) | 2.05 (1.09-3.84)*  1.94 (1.02-3.66)*  2.13 (1.12-4.02)*  2.02 (1.07-3.79)*  2.06 (1.09-3.87)*  2.03 (1.08-3.82)*  1.99 (1.05-3.74)*  1.87 (0.99-3.54)  2.14 (1.09-4.24)* |
|  | *E/B ratio* (Crude OR)  Adjusted for location  Adjusted for maternal race  Adjusted for maternal asthma during pregnancy  Adjusted for maternal allergy during pregnancy  Adjusted for type of home  Adjusted for moving home  Adjusted for present of siblings  Adjusted for breastfeeding status-3 months | 0.92 (0.30-2.89)  0.95 (0.30-3.01)  0.82 (0.26-2.63)  0.93 (0.30-2.90)  0.90 (0.28-2.82)  0.92 (0.29-2.89)  0.96 (0.30-3.02)  0.96 (0.30-3.02)  0.92 (0.29-2.91) | 0.47 (0.25-0.89)*  0.46 (0.24-0.87)*  0.44 (0.23-0.84)*  0.47 (0.25-0.89)*  0.46 (0.24-0.88)*  0.47 (0.25-0.89)*  0.50 (0.26-0.94)*  0.49 (0.26-0.93)*  0.46 (0.24-0.87)* |
|  | *Bacteroidaceae* (Crude OR)  Adjusted for location  Adjusted for maternal race  Adjusted for maternal asthma during pregnancy  Adjusted for maternal allergy during pregnancy  Adjusted for type of home  Adjusted for moving home  Adjusted for present of siblings  Adjusted for breastfeeding status-3 months | 1.52 (0.48-4.82)  1.47 (0.46-4.70)  1.67 (0.52-5.46)  1.52 (0.48-4.81)  1.61 (0.50-5.15)  1.51 (0.47-4.79)  1.48 (0.46-4.70)  1.45 (0.45-4.61)  1.55 (0.49-4.93) | 2.17 (1.15-4.09)*  2.19 (1.16-4.15)*  2.30 (1.21-4.38)*  2.16 91.15-4.08)*  2.34 (1.23-4.47)*  2.17 (1.15-4.08)*  2.06 (1.09-3.91)*  2.07 (1.09-3.95)*  2.24 (1.18-4.28)* |
|  | *Streptococcaceae* (Crude OR)  Adjusted for location  Adjusted for maternal race  Adjusted for maternal asthma during pregnancy  Adjusted for maternal allergy during pregnancy  Adjusted for type of home  Adjusted for moving home  Adjusted for present of siblings  Adjusted for breastfeeding status-3 months | 0.20 (0.06-0.70)*  0.21 (0.06-0.75)*  0.20 (0.56-0.72)*  0.20 (0.06-0.70)*  0.20 (0.06-0.71)*  0.20 (0.06-0.72)*  0.20 (0.06-0.71)*  0.20 (0.06-0.70)*  0.19 (0.05-0.66)* | 0.31 (0.16-0.58)*  0.31 (0.16-0.59)*  0.30 (0.16-0.58)*  0.31 (0.16-0.59)*  0.30 (0.16-0.58)*  0.31 (0.16-0.59)*  0.31 (0.16-0.59)*  0.30 (0.15-0.57)*  0.29 (0.15-0.56)* |
|  | *Ruminococcus* (Crude OR)  Adjusted for location  Adjusted for maternal race  Adjusted for maternal asthma during pregnancy  Adjusted for maternal allergy during pregnancy  Adjusted for type of home  Adjusted for moving home  Adjusted for present of siblings  Adjusted for breastfeeding status-3 months | 1.41 (0.44-4.51)  1.37 (0.43-4.43)  1.11 (0.34-3.64)  1.41 (0.44-4.51)  1.41 (0.44-4.50)  1.38 (0.43-4.44)  1.40 (0.44-4.46)  1.41 (0.44-4.53)  1.32 (0.40-4.37) | 2.43 (1.29-4.59)*  2.40 (1.27-4.55)*  2.26 (1.17-4.33)*  2.43 (1.28-4.59)*  2.33 (1.23-4.42)*  2.42 (1.28-4.58)*  2.38 (1.26-4.53)*  2.34 (1.23-4.47)*  2.39 (1.23-4.64)* |
| Caesarean-scheduled | *Clostridium* (Crude OR)  Adjusted for location  Adjusted for maternal race  Adjusted for maternal asthma during pregnancy  Adjusted for maternal allergy during pregnancy  Adjusted for type of home  Adjusted for moving home  Adjusted for present of siblings  Adjusted for breastfeeding status-3 months | 3.00 (0.29-31.23)  4.29 (0.38-48.83)  2.91 (0.28-30.45)  3.10 (0.30-32.34)  2.28 (0.21-24.39)  3.12 (0.30-32.71)  2.97 (0.29-31.00)  2.83 (0.23-34.49)  3.28 (0.31-35.10) | 2.73 (1.09-6.83)*  3.25 (1.20-8.80)*  2.56 (0.97-6.76)  2.64 (1.05-6.65)*  2.65 (1.04-6.75)*  2.73 (1.09-6.85)*  2.74 (1.09-6.88)*  3.01 (1.14-7.91)*  2.86 (1.13-7.24)* |
|  | *Oscillospira* (Crude OR)  Adjusted for location  Adjusted for maternal race  Adjusted for maternal asthma during pregnancy  Adjusted for maternal allergy during pregnancy  Adjusted for type of home  Adjusted for moving home  Adjusted for present of siblings  Adjusted for breastfeeding status-3 months | 0.67 (0.06-7.01)  0.59 (0.06-6.41)  0.61 (0.05-6.95)  0.72 (0.07-7.55)  0.72 (0.07-7.84)  0.64 (0.06-6.78)  0.63 (0.06-6.66)  0.92 (0.08-11.18)  0.42 (0.03-5.62) | 3.13 (1.27-7.67)*  3.01 (1.18-7.65)*  2.59 (0.98-6.82)  3.39 (1.36-8.48)*  3.37 (1.36-8.35)*  3.14 (1.28-7.71)*  3.33 (1.33-8.30)*  3.13 (1.25-7.83)*  4.28 (1.52-12.03)* |
| Caesarean-emergency | *Chao1_Proteobacteria* (Crude OR)  Adjusted for location  Adjusted for maternal race  Adjusted for maternal asthma during pregnancy  Adjusted for maternal allergy during pregnancy  Adjusted for type of home  Adjusted for moving home after birth  Adjusted for present of siblings  Adjusted for breastfeeding status-3 months | 0.49 (0.12-2.02)  0.50 (0.12-2.11)  0.49 (0.12-2.05)  0.43 (0.10-1.83)  0.48 (0.11-1.99)  0.47 (0.11-1.97)  0.46 (0.11-1.93)  0.48 (0.11-2.11)  0.47 (0.11-1.95) | 0.26 (0.11-0.63)*  0.27 (0.11-0.66)*  0.26 (0.10-0.65)*  0.24 (0.10-0.59)*  0.27 (0.11-0.65)*  0.25 (0.10-0.63)*  0.26 (0.11-0.62)*  0.20 (0.07-0.52)*  0.25 (0.10-0.61)* |
|  | *Bifidobacteriaceae* (Crude OR)  Adjusted for location  Adjusted for maternal race  Adjusted for maternal asthma during pregnancy  Adjusted for maternal allergy during pregnancy  Adjusted for type of home  Adjusted for moving home  Adjusted for present of siblings  Adjusted for breastfeeding status-3 months | 7.53 (1.44-39.50)*  8.09 (1.51-43.30)*  8.38 (1.55-45.36)*  7.43 (1.40-39.50)*  7.52 (1.43-39.53)*  8.13 (1.52-43.65)*  8.66 (1.58-47.5)*  8.23 (1.55-43.72)*  8.52 (1.56-46.66)* | 1.67 (0.74-3.75)  2.00 (0.84-4.75)  1.81 (0.75-4.37)  1.65 (0.73-3.76)  1.81 (0.79-4.16)  1.83 (0.77-4.38)  1.71 (0.75-3.88)  1.93 (0.80-4.63)  1.84 (0.79-4.28) |
|  | *Ruminococcus* (Crude OR)  Adjusted for location  Adjusted for maternal race  Adjusted for maternal asthma during pregnancy  Adjusted for maternal allergy during pregnancy  Adjusted for type of home  Adjusted for moving home  Adjusted for present of siblings  Adjusted for breastfeeding status-3 months | 0.52 (0.10-2.71)  0.52 (0.10-2.78)  0.47 (0.09-2.52)  0.51 (0.10-2.70)  0.52 (0.10-2.74)  0.62 (0.11-3.38)  0.54 (0.10-2.86)  0.52 (0.10-2.79)  0.34 (0.05-2.10) | 2.53 (1.11-5.75)*  2.50 (1.05-5.91)*  2.21 (0.93-5.25)  2.50 (1.09-5.74)*  2.55 (1.10-5.88)*  3.38 (1.35-8.46)*  2.58 (1.13-5.88)*  2.72 (1.11-6.64)*  2.04 (0.84-4.94) |
|  | *Oscillospira* (Crude OR)  Adjusted for location  Adjusted for maternal race  Adjusted for maternal asthma during pregnancy  Adjusted for maternal allergy during pregnancy  Adjusted for type of home  Adjusted for moving home  Adjusted for present of siblings  Adjusted for breastfeeding status-3 months | 0.81 (0.19-3.53)  0.81 (0.18-3.55)  0.67 (0.15-3.03)  0.82 (0.19-3.66)  0.81 (0.19-3.54)  0.97 (0.21-4.39)  0.84 (0.19-3.18)  0.75 (0.16-3.44)  0.60 (0.12-2.94) | 3.54 (1.54-8.14)*  3.74 (1.56-8.97)*  2.74 (1.15-6.55)*  3.59 (1.54-8.35)*  3.67 (1.57-8.60)*  4.64 (1.84-11.71)*  3.61 (1.56-8.32)*  2.78 (1.14-6.80)*  3.08 (1.27-7.49)* |
|  | *Unclassified Enterobacteriaceae* (Crude OR)  Adjusted for location  Adjusted for maternal race  Adjusted for maternal asthma during pregnancy  Adjusted for maternal allergy during pregnancy  Adjusted for type of home  Adjusted for moving home  Adjusted for present of siblings  Adjusted for breastfeeding status-3 months | 0.36 (0.09-1.45)  0.36 (0.09-1.47)  0.41 (0.10-1.67)  0.32 (0.08-1.33)  0.36 (0.09-1.45)  0.37 (0.09-1.53)  0.34 (0.08-1.39)  0.40 (0.10-1.60)  0.39 (0.10-1.63) | 0.35 (0.15-0.81)*  0.36 (0.14-0.82)*  0.42 (0.18-1.02)  0.32 (0.13-0.77)*  0.36 (0.15-0.85)*  0.36 (0.15-0.89)*  0.34 (0.15-0.80)*  0.38 (0.15-0.92)*  0.39 (0.16-0.93)* |

OR, odds ratio; aOR, adjusted odds ratio; CI, confidence interval; F/P, Firmicutes/Proteobacteria; E/B, Enterobacteriaceae/Bacteroidaceae; Richness and diversity indices measured at OTU level.

*P<0.05

^#^Any antibiotic exposure of infants before microbiota sampling at 3 months including both indirect exposure (maternal IAP) and direct exposure (IV and oral antibiotics)
