# Supplementary material for: Exposure to household furry pets influences the gut microbiota of infant at 3–4 months following various birth scenarios
Source: Microbiome. 2017 Apr 6;5:40. doi: 10.1186/s40168-017-0254-x (PMC5382463; doi:10.1186/s40168-017-0254-x)
Supplement: Supplementary file 6 — Linear discriminant analysis (LDA) scores for differentially abundant bacterial taxa due to pet exposure in formula-fed infants born by Caucasian mothers without prior direct antibiotic exposure until 3 months old following different birth scenarios (P < 0.05). (DOCX 54 kb) [file 40168_2017_254_MOESM6_ESM.docx]

**Table S6. Linear discriminant analysis (LDA) scores for differentially abundant of bacterial taxa due to pet exposure in formula-fed infants born by Caucasian mothers without prior direct antibiotic exposure until 3 months old following different birth scenarios (P<0.05).**

| **Birth Scenarios** | **Taxa**  **(Taxon level_Name)** | **Discriminative Factor** | **LDA Scores^*^** |
| --- | --- | --- | --- |
| Vaginal, IAP- | f__Ruminococcaceae | Only Prenatal | 4.2 |
| Vaginal, IAP+ | g__Bilophila | No exposure | 3.5 |
| Caesarean-Emergency | g_Unclssified Ruminococcaceae | Both Pre and Postnatal | 4.0 |
|  | g__Citrobacter | No exposure | 3.7 |
|  | g__Lactococcus | No exposure | 2.1 |

*Logarithmic LDA (Linear Discriminant Analysis) score is a confidence score for a taxa that would be predicted under certain discriminative factor (pet exposure episodes).

In this analysis, children were unified by excluding infants from non-Caucasian mothers, also experienced antibiotic exposure from birth to 3 months old (for vaginally born infants without received IAP), and received exclusively maternal breast milk.
