# Supplementary material for: Exposure to household furry pets influences the gut microbiota of infant at 3–4 months following various birth scenarios
Source: Microbiome. 2017 Apr 6;5:40. doi: 10.1186/s40168-017-0254-x (PMC5382463; doi:10.1186/s40168-017-0254-x)
Supplement: Supplementary file 7 — Pet exposure associated changes in gut microbiota of selected infants from Caucasian mothers with no anitobiotic exposure and no exclusi ve breastfeeding at 3 months following different birth scenarios. (PDF 839 kb) [file 40168_2017_254_MOESM7_ESM.pdf]

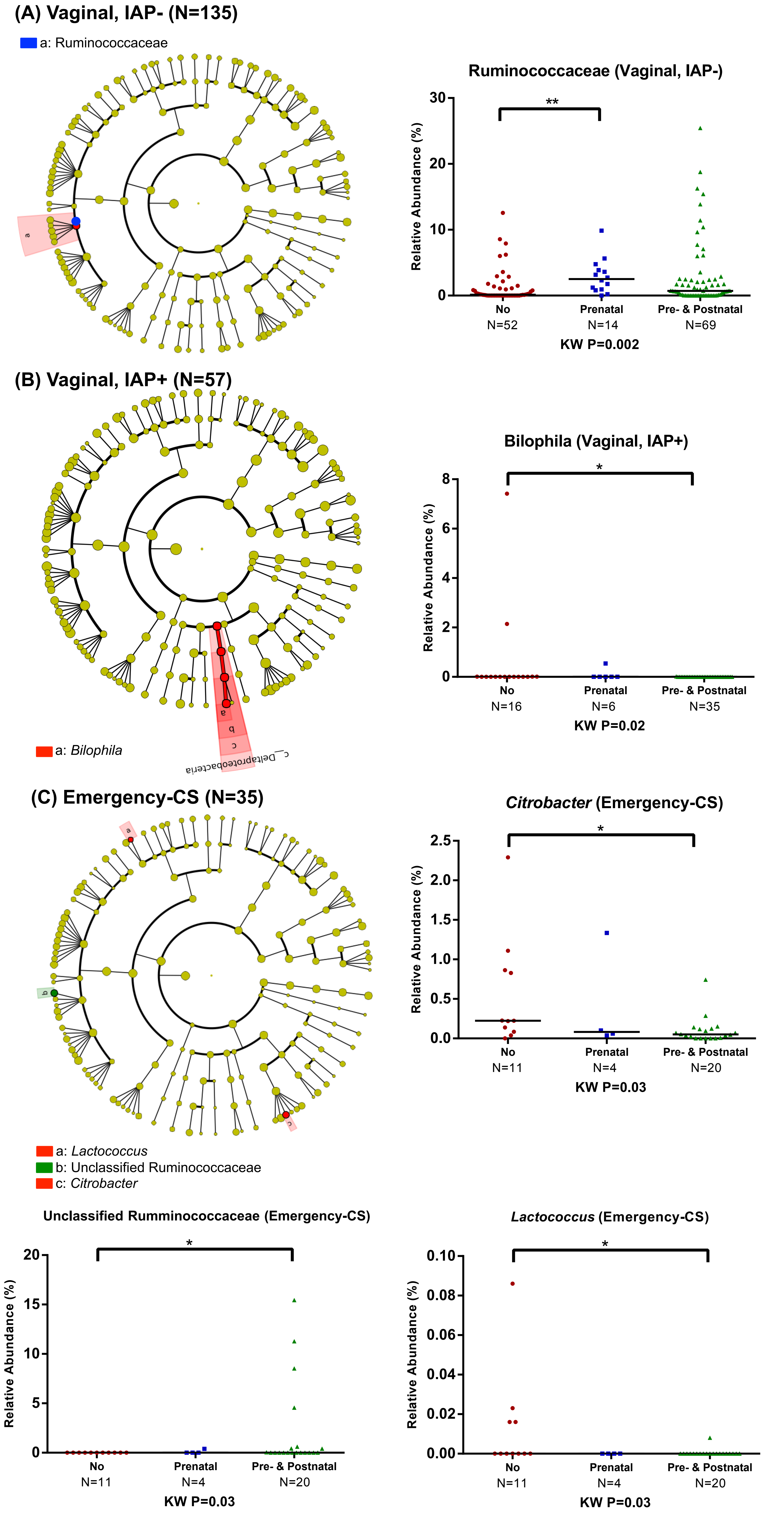

(A) Vaginal, IAP- (N=135)
a: Ruminococcaceae
a
Ruminococcaceae (Vaginal, IAP-)
Relative Abundance (%)
30
20
10
0
**
No
N=52
Prenatal
N=14
Pre- & Postnatal
N=69
KW P=0.002
(B) Vaginal, IAP+ (N=57)
a
b
c
c__Deltaproteobacteria
a: Bilophila
Bilophila (Vaginal, IAP+)
Relative Abundance (%)
8
6
4
2
0
*
No
N=16
Prenatal
N=6
Pre- & Postnatal
N=35
KW P=0.02
(C) Emergency-CS (N=35)
a
b
c
a: Lactococcus
b: Unclassified Ruminococcaceae
c: Citrobacter
Citrobacter (Emergency-CS)
Relative Abundance (%)
2.5
2.0
1.5
1.0
0.5
0.0
*
No
N=11
Prenatal
N=4
Pre- & Postnatal
N=20
KW P=0.03
Unclassified Rumminococcaceae (Emergency-CS)
Relative Abundance (%)
20
15
10
5
0
*
No
N=11
Prenatal
N=4
Pre- & Postnatal
N=20
KW P=0.03
Lactococcus (Emergency-CS)
Relative Abundance (%)
0.10
0.08
0.06
0.04
0.02
0.00
*
No
N=11
Prenatal
N=4
Pre- & Postnatal
N=20
KW P=0.03
