# Supplementary material for: Exposure to household furry pets influences the gut microbiota of infant at 3–4 months following various birth scenarios
Source: Microbiome. 2017 Apr 6;5:40. doi: 10.1186/s40168-017-0254-x (PMC5382463; doi:10.1186/s40168-017-0254-x)
Supplement: Supplementary file 8 — Relative abundance of dominant genera in faecal microbiota of infants belonged to different stratified groups, according to birth scenarios and pet exposure. (DOCX 102 kb) [file 40168_2017_254_MOESM8_ESM.docx]

**Table S7**. **Relative abundance of dominant genera in fecal microbiota of infants belonged to different stratified groups, according to birth scenarios and pet exposure.**

| **Birth Scenarios** | **Dominant Genera^#^** | **No Siblings** | | | | **No Exclusive Breastfeeding** | | | | **No Siblings and No Exclusive Breastfeeding** | | | |
| --- | --- | --- | --- | --- | --- | --- | --- | --- | --- | --- | --- | --- | --- |
|  |  | **No exposure** | **Only prenatal** | **Both pre and postnatal** | **P** | **No exposure** | **Only prenatal** | **Both pre and postnatal** | **P** | **No exposure** | **Only prenatal** | **Both pre and postnatal** | **P** |
|  |  | 162 (42.6%)  Median  (IQR) | 29 (7.6%)  Median  (IQR) | 189 (49.8%)  Median (IQR) |  | 147 (41.4%)  Median  (IQR) | 32 (9.0%)  Median  (IQR) | 176 (49.6%)  Median  (IQR) |  | 70 (38.7%)  Median  (IQR) | 12 (6.6%)  Median  (IQR) | 99 (54.7%)  Median (IQR) |  |
| Vaginal IAP- | *Unclassified Lachnospiraceae* | 0.02  (0-2.68) | 0.12  (0.01-1.8)* | 0.04  (0.01-1.2) | 0.44 | 0.13  (0.01-3.54) | 4.3  (0.41-7.8)* | 0.68  (0.03-2.9) | 0.03 | 0.83  (0.01-7.5) | 5.5  (1.01-8.0) | 0.68  (0.03-2.9) | 0.07 |
|  | *Ruminococcus* | 0.01  (0-0.3) | 0.12  (0-1.7)** | 0.02  (0-1.8)** | 0.11 | 0.03  (0-1.6) | 1.1  (0.23-4.5)** | 0.3  (0.01-3.5) | 0.01 | 0.03  (0-1.5) | 1.0  (0.05-2.2) | 0.3  (0.01-2.7) | 0.1 |
|  | *Oscillospira* | 0  (0-0.01) | 0  (0-1.4)** | 0.01  (0-0.71)** | 0.01 | 0  (0-0.64) | 0.81  (0-3.4)** | 0.41  (0-1.5) | 0.002 | 0  (0-1.7) | 0.73  (0-3.4) | 0.42  (0-1.5) | 0.04 |
|  | *Unclassified Enterobacteriaceae* | 19.1  (10.8-34.6) | 6.2  (3.1-27.8)** | 13.9  (4.6-34.2) | 0.05 | 12.6  (5.6-30.1) | 1.6  (0.85-8.6)** | 9.5  (4.1-20.7) | 0.002 | 12.2  (5.7-30.8) | 1.4  (0.74-10.4)* | 8.1  (3.8-15.8) | 0.01 |
| Vaginal IAP+ | *Ruminococcus* | 0  (0-0.67) | 0.02  (0-1.1) | 0.03  (0.01-1.9)** | 0.03 | 0.06  (0-1.3) | 0.02  (0-0.02) | 0.4  (0.01-2.0) | 0.07 | 0.42  (0-1.4) | 0.02  (0-1.2) | 0.48  (0.01-2.0) | 0.18 |
| Caesarean-emergency | *Parabacteroides* | 0  (0-0.01) | 0.02  (0-0.04) | 0  (0-0.01) | 0.15 | 0  (0-0.01) | 0.01  (0-22.3) | 0  (0-0.02) | 0.49 | 0  (0-0.02) | 0  (0-33.5) | 0  (0-0.02) | 0.84 |
|  | *Ruminococcus* | 0  (0-0.03) | 0  (0-0.01) | 0.04  (0-4.6)* | 0.04 | 0.02  (0-7.9) | 0.01  (0-2.0) | 1.5  (0.01-7.2) | 0.47 | 0.02  (0.01-5.3) | 0.65  (0-2.3) | 1.5  (0.01-7.2) | 0.56 |
|  | *Oscillospira* | 0.01  (0-0.87) | 0  (0-1.2) | 0.35  (0.01-2.8) | 0.05 | 0.21  (0.01-1.2) | 0.01  (0-4.7) | 0.72  (0.11-3.7) | 0.36 | 0.45  (0.01-2.1) | 0.57  (0-6.5) | 0.72  (0.11-3.7) | 0.8 |

IQR, interquartile rage.

*Dominant genera have overall median relative abundance >1% at 3-4 months; phyla are in the plain text and families are italized. Comparisons by nonparametric Kruskal-Wallis test.

Post-hoc comparisons between no exposure group and either group of exposure were done by Mann-Whitney U test. * *P<0.05*, ***P<0.01*, ****P<0.0001*.
